# Influence of gRNA efficiency and inversion size on the frequency of CRISPR/Cas9-induced chromosomal inversions in tomato protoplasts

**DOI:** 10.1186/s12870-026-08442-9

**Published:** 2026-03-10

**Authors:** Jillis Grubben, Gerard Bijsterbosch, Richard G.F. Visser, Henk J. Schouten

**Affiliations:** 1 Department of Plant Breeding, Wageningen University & Research, Wageningen, The Netherlands; 2 Graduate School Experimental Plant Sciences, Wageningen University & Research, Wageningen, The Netherlands

**Keywords:** CRISPR-Cas9, Chromosomal inversions, Tomato (*Solanum lycopersicum*), Protoplast transfection, Chromosome engineering, Non-Homologous End-Joining (NHEJ), Digital PCR (dPCR), Long-read sequencing (PacBio)

## Abstract

**Background:**

Clustered Regularly Interspaced Short Palindromic Repeats (CRISPR)-Cas9 enables induction of chromosomal inversions from hundreds of base pairs to millions of base pairs, but the factors influencing inversion frequency are not well understood. Prior reports differ in species, detection methods, and delivery strategies, making direct comparisons difficult. We addressed this by introducing a normalisation strategy based on a reference guide RNA (gRNA) as an internal standard and testing inversion sizes spanning kilobases to tens of megabases in tomato protoplasts.

**Results:**

Tomato (*Solanum lycopersicum*) protoplasts were transfected with constructs encoding a fixed “reference” gRNA and a second “variable” gRNA positioned at increasing genomic distances, creating potential inversions from 1 kilobase to 37.5 megabases. Using the reference gRNA to normalise across samples, we found that up to ~ 1 megabase, inversion frequency tracked the cutting efficiency of the less efficient gRNA, consistent with gRNA activity being a major contributor within the chromosome tested. For these intervals, the inversion frequencies reached up to 1.24% when both gRNAs were efficient. Above ~ 1 megabase, inversion frequencies declined sharply despite efficient cutting, suggesting a size-dependent barrier to inversion formation; for example, 37.5 megabase inversions occurred at substantially lower frequency (up to 0.18%) despite efficient gRNAs. Because each interval corresponds to a distinct genomic location. Inversions were only observed when both gRNAs were active, and large deletions were more frequent than inversions when dual breaks were induced.

**Conclusions:**

In our experiments, the gRNA cutting efficiency was a major determinant of inversion frequency in our experiment up to ~ 1 megabase, while locus-specific genomic context may also contribute., Larger inversions may be limited by an additional, size-dependent constraint. These findings inform the design of edits aimed at reverting breeding-relevant inversions (for example, those linked to resistance loci) and suggest that achieving high efficiency for multi-megabase inversions will require strategies that overcome spatial or repair-related constraints. The internal reference gRNA normalises sample-to-sample variability in DNA delivery and Cas9 activity, enabling direct comparison of the performance of different gRNAs across samples on a shared, ratio-based scale. This ratio-to-reference strategy may likewise be used to benchmark gRNA performance and edit yields (inversions, deletions, translocations, and base/prime edits) across transfections in plant protoplasts, and may be extended to additional cell systems beyond plants.

**Supplementary Information:**

The online version contains supplementary material available at 10.1186/s12870-026-08442-9.

## Background

Genomic rearrangements, including inversions, are significant sources of genetic diversity among related species [1]. An inversion is a type of chromosomal rearrangement where a segment of the chromosome is reversed in orientation, such that the gene order within the segment is inverted [2]. Inversions can vary greatly in size, with reports ranging from tens of base pairs to hundreds of kilobase pairs [3, 4]. Inversion events can occur naturally and may become fixed in a population, potentially causing fitness effects, reduced meiotic recombination, and even speciation events [5]. Two mechanisms have been proposed for inversion formation: spontaneous excision and inversion of a genomic region between two transposons, followed by re-ligation into the genome [6], and simultaneous double-strand breaks in a chromosome, which are then repaired via canonical non-homologous end-joining (c-NHEJ) in an inverted orientation [7]. During meiosis, inversions in heterozygotes may pair through inversion loops, but crossovers within these loops can cause the loss of large chromosomal sections, leading to lethal effects in the progeny [8]. Consequently, crossovers in inversions are rare among organisms heterozygous for the inversion [9, 10]. This rarity makes it challenging for plant breeders to eliminate linkage drag from an inversion if a target gene is located within that region.

Several resistance genes in wild tomato relatives are located within inversions compared to the reference genome. The *Ty-2* gene on chromosome 11 in *Solanum habrochaites* confers resistance to tomato yellow leaf curl virus, while the *Mi-1* gene on chromosome 6 in *S. peruvianum* confers resistance to root-knot nematodes [11, 12]. These genes are located within 200 kbp and 300 kbp inversions, respectively [3, 13]. These inversions prevent recombination with homologous chromosomal segments in tomato varieties [14]. Consequently, all modern tomato varieties harbouring the *Mi-1* and/or *Ty-*2 genes carry linkage drag from the wild donors. This issue in plant breeding has not been solved yet, although it exists already for decades [9].

These linkage drag challenges caused by inversions can be overcome by harnessing genome editing techniques such as CRISPR/Cas9. Researchers have induced inversions using CRISPR/Cas9 by generating two DSBs on the same chromosome. When two DSBs are generated, an inversion can be formed when the excised fragment is ligated back in the opposite orientation through c-NHEJ [15]. This technique was used to revert an inversion of 1.1 Mbp. Both normal chromosome pairing during meiosis and recombination in the reverted region were re-established after reverting the inversion [16]. In addition to reversing inversions, new inversions have also been generated to re-shuffle promoter-gene combinations. This involves excising DNA segments between two promoter-gene borders on the same chromosome, as demonstrated in rice where a heritable inversion of 911 kbp was induced [17]. Further, researchers have shown that recombination can be suppressed between favourable genes physically bound together in a cluster on a chromosome. Recombination was suppressed by inverting 90% of chromosome 2 in *A. thaliana* [18]. The CRISPR/Cas9 system has been used to induce targeted inversions in a variety of species successfully, and various inversion sizes and induction frequencies have been presented [17, 19–22]. For instance, Zhang et al. (2017) achieved a 2.6% efficiency for a 341 bp inversion in *A. thaliana* [19], while Schmidt et al. (2019) used floral dip to generate inversions ranging from 2.9 to 18 kbp at an induction frequency of 1% [21]. In rice, a 911 kbp inversion occurred in three out of 259 calli, and in maize, a significant 75.5 Mbp inversion was found in two out of 1,500 embryos [17, 22].

Recent advances have enabled the generation of inversions of various sizes, ranging from hundreds of bases to tens of megabases, in multiple species with no apparent size limit. However, the size range of inversions reported within these studies often falls within a single order of magnitude and presents a limited number of inversion events. Moreover, these studies were based on different species, were using different detection methods and were using different transduction systems. Additionally, no studies have examined the relationship between inversion frequency and size. This makes it impossible to systematically investigate the relative contributions of gRNA cutting efficiency and inversion size to CRISPR/Cas9-induced inversion frequency from these studies. The goal of our research was to investigate how inversion length and CRISPR/Cas9 cutting efficiency together influence the frequency of CRISPR/Cas9-induced inversions. For this purpose, we induced a series of inversions, ranging from 1 kbp to 37.5 Mbp in chromosome 6 of *Solanum lycopersicum*. The latter inversion covered approximately three-quarters of the chromosome including the centromere. Our results show a strong association between gRNA cutting frequency and inversion frequency. Within our tested intervals smaller than 1 Mbp, we did not observe a consistent decline in inversion frequency with increasing distance, but locus-specific effects cannot be excluded. We therefore cannot exclude that the 1 Mb interval reflects locus-specific properties. However, inversions larger than 1 Mbp had much lower frequencies, despite high gRNA-cutting activity. We discuss a model that may explain these observations.

## Methods

### CRISPR/Cas9 construct generation

To generate inversions of different sizes, CRISPR/Cas9 constructs were created that contained two gRNAs targeting chromosome 6. The first gRNA, referred to as the ‘fixed’ gRNA, was included in all constructs and served as a baseline for normalisation in subsequent analyses. The second set of ‘variable’ gRNAs targeted regions at specified distances from the ‘fixed’ gRNA, specifically 1 kbp, 3 kbp, 10 kbp, 30 kbp, 100 kbp, 300 kbp, 1 Mbp, 3 Mbp, 10 Mbp, 30 Mbp, and 37.5 Mbp. All gRNAs were designed using CRISPOR (http://crispor.tefor.net/). gRNAs were synthesized (MacroGen Europe) and *BsaI* restriction sites were added via Phusion PCR. The gRNAs were inserted into LVL1 Golden Gate vectors containing the U6-26 promoter via cut-ligation using *BsaI* and T4 ligase and subsequently, these gRNAs were recombined with LVL 1 constructs containing pUBI::Cas9, NosP::NPTII, and pCsVMV::turboGFP (Supplementary Table 2, Supplementary Fig. 3). Plasmids were isolated using the Plasmid Midi Kit (Qiagen).

### Plant material and growth conditions

Wild-type *Solanum lycopersicum* cv. Moneymaker seeds were sterilized in 1% NaClO for 20 min and washed in sterilized MilliQ. The sterilised seeds were sown on a germination medium consisting of ½ MS (including vitamins from Duchefa), 3% sucrose, and 0.8% Daishin agar (pH 5.8). Seeding was performed in sterile tissue culture vessels (OS140BOX/green filter, Duchefa), with each vessel containing 10 seeds. The plants were subsequently grown in vitro at 24 °C, with 60% relative air humidity and a light intensity of 150 W/m², within propagation containers for one month.

### Protoplast generation and isolation

Protoplast transfection was conducted under sterile conditions, following a protocol adapted from Maas & Werr [23]. Approximately 20 small leaves from one-month-old seedlings were collected and finely sliced in a feather-like pattern from the midrib to the leaf edge. These were then placed abaxial side down in a Petri dish with 10 mL of digestion solution (0.4 M mannitol, 20 mM MES, 20 mM KCl, 10 mM CaCl2.2H2O, pH 5.7) for an initial wash. This digestion solution was removed, and 20 mL of freshly prepared, filter-sterilised digestion solution containing 1% cellulase R10 and 0.3% Macerozym R10 enzymes (Duchefa) was added. After incubating for 17 h in the dark at 25 °C to facilitate cell wall digestion, the digested leaf material was transferred through a Falcon 100 μm cell strainer (Corning). To the resulting cell suspension, 10 mL of W5 washing buffer (154 mM NaCl, 125 mM CaCl_2_.2H_2_O, 5 mM KCl, and 2 mM MES, pH 5.7) was added. This suspension was then centrifuged (Eppendorf 5810R) at 100 RCF for 3 min at room temperature to pellet the protoplasts. The protoplasts were resuspended in 10 mL of fresh W5 solution and centrifuged again under the same conditions. Finally, the protoplasts were resuspended in 10 mL of MMg solution (0.4 M Mannitol, 15 mM MgCl2, and 4 mM MES, pH 5.7). After a 3-minute centrifugation at 100 RCF, the protoplasts were resuspended again in 10 mL of MMg. The density of the protoplasts was determined using a Fuchs-Rosenthal haemocytometer and adjusted to a density of 1 million protoplasts per mL using MMg solution in preparation for transfection.

### Protoplast transfection

Per sample, 10 µg of plasmid (final volume adjusted to 20 µL MilliQ) was added to a 2 mL tube (Eppendorf). Next, 200 µL of the protoplast suspension was carefully pipetted onto the plasmid using pipette tips with a widened orifice. Subsequently, 200 µL freshly prepared PEG solution (4.0 g PEG-4000 (Fluka), 3.0 mL MilliQ, 2.5 mL 0.8 M mannitol solution, and 1.0 mL 1 M CaCl_2_ solution) was added and the suspension was gently but thoroughly mixed and incubated for 15 min. Subsequently, 500 µL of W1 buffer (0.5 M Mannitol, 20 mM KCl, and 4 mM MES, pH 5.7) was added droplet-wise to the suspension, after which the process was repeated for the next sample. Another 500 µL of W1 was added to each sample, and the samples were then centrifuged at 200 RCF for 3 min. The supernatant was then removed by pipetting and 1 mL of W1 was added to the protoplasts as described above. Samples were centrifuged, supernatant was removed, and protoplasts were resuspended in 150 µL W1. Protoplasts were incubated in the dark for 48 h at 25 °C. Transfection efficiency was determined by measuring the ratio of fluorescent (GFP-positive) to surviving protoplasts under an Axio Vert.A1 Inverted Microscope (Carl Zeiss™) for quality control purposes. Centrifugation of protoplasts was performed at 200 RCF. Following centrifugation, the supernatant was discarded, and the remaining protoplasts were immediately flash-frozen in liquid nitrogen for long-term storage.

### DNA isolation and purification

DNA was isolated from protoplasts using the NucleoMag Plant^®^ kit combined with the KingFisher Flex System (Thermo Scientific™), following the manufacturers’ protocol. The DNA was eluted in 50 µL of Milli-Q water. We noted that traces from the NucleoMag Plant^®^ kit negatively impacted the crystal matrix formation in cdPCR in our experiments. This issue was addressed by purifying the isolated DNA with a NaCl/ethanol-based wash. To each 50 µL DNA sample, 1 µL of 5 µg/µL molecular grade glycogen (Thermo Scientific™) was added as a carrier. This was followed by the addition of 5 µL of 5 M NaCl and 55 µL of isopropanol. The samples were then stored at -20 °C for 24 h to precipitate DNA. Subsequently, samples were centrifuged at 20,000 g for one hour at 4 °C. The supernatant was carefully removed without disturbing the DNA pellet. The pellet was then washed by adding 100 µL of 70% ethanol followed by another centrifugation step at 20,000 g for 30 min at 4 °C. Subsequently, the ethanol was removed, and the DNA pellet was dried at 37 °C for 2 min to remove residual ethanol. Finally, the DNA was diluted in 15 µL Milli-Q water and DNA concentrations were measured using the Qubit High Sensitivity kit (Invitrogen™).

### Detecting inversions with Sanger sequencing

Isolated DNA was enriched for inversion events at both DSB sites through separate PCR reactions using Phusion™ High-Fidelity DNA Polymerase (Thermo Scientific™) following the manufacturer’s protocol. A pair of inversion-specific primers were used for each DSB border, each oriented in reverse to the wild-type DNA (Fig. 2a, Supplementary Table 3). The amplicons harbouring the inversion borders were cloned into *Escherichia coli* via Golden Gate cloning. Plasmids were isolated using the QIAprep Spin Miniprep Kit (Qiagen) from cell cultures derived from single colonies. Subsequently, Sanger sequencing (Macrogen Europe) was used to obtain the DNA sequences containing the inversion borders that were used for analysis.

### Composition of cdPCR master mix and probe design

The quantification of inversions was performed using Crystal Digital PCR™ technology, in combination with the Geode automatic droplet generator and the Prism3 multi-colour fluorescence imager. The Crystal Miner software v3.0 7 3 manual was followed and minor adjustments were made to optimise the analysis of inversion events. The cdPCR master mix consisted of 5 µL of Perfecta Multiplex qScript ToughMix 5X polymerase (Quantabio), 2.5 µL of 100 nM fluorescin, 11 µL of DNA sample, and 1 µL of 25 µM inversion-specific primers, and 6.25 µM of FAM and Cy-5 labelled TaqMan ZEN™ double quenched probes (Integrated DNA Technologies; Fig. 2a, Supplementary Table 3). Nuclease-free water was added to reach a final volume of 25 μL. FAM and Cy-5 dyes were selected due to their well-separated emission spectra to minimise spectral crosstalk between the probes. The cdPCR inversion detection method was specifically arranged with the FAM-labelled probe targeting one side of the inversion border and the Cy-5 labelled probe targeting the opposite border. This arrangement ensured that fluorescence signals from both probes were emitted only in the presence of the expected sequences at each inversion border (Fig. 2a, b). The expected sequences for each targeted inversion were synthesised as gBlocks™ (Integrated DNA Technologies) and are listed in Supplementary Table 4. These gBlocks were used as positive controls and to facilitate the calculation of spillover compensation matrices between the fluorescence detection channels.

The used parameters of the cdPCR Naica Geode and Prism3 system were adapted from those described by [24] with minor adjustments made to the 2-step PCR thermal cycling program to optimise amplicon generation. The adjusted protocol included an initial denaturation step at 95 °C for 10 min, followed by 45 cycles of denaturation at 95 °C for 30 s and annealing at 60 °C for 15 s. The preset FAM and Cy-5 excitation wavelengths were selected and the exposure times for FAM and Cy-5 were set to 65 ms and 50 ms, respectively.

The partitioned PCR reactions were inspected using the “Quality Control” section in the Crystal Reader software and samples with fewer than 18,000 partitioned PCR reactions were excluded from analysis. Inversion events in partitioned PCR reactions were counted when both the FAM and Cy-5 channels registered signals above the threshold and flagged by the software as positive events (Fig. 2b). It was manually confirmed that these individual positive partitioned PCR reactions exceeded the background signal threshold in both channels, using the ‘Quality Control’ section of the Crystal Reader software.

### Estimating genome copy number and calculating inversion frequency

The genome copy number that was added per cdPCR chip slot was calculated using the equation below and the total tomato genome size of 1,179 Mbp [25].

Equation 1.

$$\:\mathrm{G}\mathrm{e}\mathrm{n}\mathrm{o}\mathrm{m}\mathrm{e}\:\mathrm{c}\mathrm{o}\mathrm{p}\mathrm{y}\:\mathrm{n}\mathrm{u}\mathrm{m}\mathrm{b}\mathrm{e}\mathrm{r}\:=\:\frac{\left(\mathrm{M}\mathrm{a}\mathrm{s}\mathrm{s}\:\mathrm{o}\mathrm{f}\:\mathrm{D}\mathrm{N}\mathrm{A}\:\mathrm{(g)}\mathrm{*}\:\mathrm{N}\mathrm{o}.\:\mathrm{A}\mathrm{v}\mathrm{o}\mathrm{g}\mathrm{a}\mathrm{d}\mathrm{r}\mathrm{o}\right)}{\left(\mathrm{G}\mathrm{e}\mathrm{n}\mathrm{o}\mathrm{m}\mathrm{e}\:\mathrm{s}\mathrm{i}\mathrm{z}\mathrm{e}\:\right(\mathrm{b}\mathrm{p}\left)\:\mathrm{*}\:\mathrm{A}\mathrm{v}\mathrm{e}\mathrm{r}\mathrm{a}\mathrm{g}\mathrm{e}\:\mathrm{b}\mathrm{a}\mathrm{s}\mathrm{e}\mathrm{p}\mathrm{a}\mathrm{i}\mathrm{r}\:\mathrm{w}\mathrm{e}\mathrm{i}\mathrm{g}\mathrm{h}\mathrm{t}\:\right(\mathrm{D}\mathrm{a}))}$$

$$\:\mathrm{G}\mathrm{e}\mathrm{n}\mathrm{o}\mathrm{m}\mathrm{e}\:\mathrm{c}\mathrm{o}\mathrm{p}\mathrm{y}\:\mathrm{n}\mathrm{u}\mathrm{m}\mathrm{b}\mathrm{e}\mathrm{r}=\:\frac{\left(1 \mathrm{*} 10^{-9} \text {*} 6.022 \text {*} 10^{23}\right)}{(\mathrm{1.179}\mathrm{*}10^9\mathrm{*}\:659.928\text)}\:$$

The genome copy number of each sample that is added to the cdPCR chip slot does not accurately reflect the number of analysed genomes. This discrepancy occurs because a portion of the sample volume does not form analysable droplets, leaving some genomes unanalysed. To address this, we used the Crystal Reader software, which automatically inspects the partitioned cdPCR droplet matrix and excludes any droplets that do not precisely match the expected volume of 0.59 nL. The total volume of the analysable droplets was then calculated by multiplying the number of qualifying droplets in each sample by 0.59 nL. This total analysable volume was then divided by the total volume added to the chip to calculate the volume fraction of the sample analysed. This volume fraction was multiplied by the total number of genomes in the sample to obtain the number of analysed genomes per sample. Finally, the inversion frequency per sample was calculated by dividing the number of detected inversions by the number of analysed genomes per sample.

### Analysis of CRISPR/Cas9 mutation induction efficiency

To assess the cutting efficiencies of the CRISPR/Cas constructs, the regions surrounding the CRISPR/Cas9 target sites were first amplified via PCR. This process used Phusion™ High-Fidelity DNA Polymerase (Thermo Scientific™) along with primers containing an eight-base-pair barcode (Supplementary Table 3). The resulting PCR amplicons were then purified using the DNA Clean & Concentrator − 5 kit (Zymo Research). After equimolar pooling, the samples were sequenced on an Illumina HiSeq system (Eurofins Europe). The sequence data were processed for demultiplexing and trimming using Geneious Prime (https://www.geneious.com/prime/), which enabled the analysis of mutations at the gRNA target sites.

CRISPR/Cas9 editing frequencies were determined from the HiSeq data by employing the “amplicanPipeline” function within AmpliCan [26] (https://github.com/valenlab/amplican; Supplementary code 1). The configuration file for Amplican specified a search window incorporating the two nucleotides at the predicted CRISPR/Cas9 cutting site, with a cut-off of 4 base pairs. For each analysed CRISPR/Cas9 site, two negative control samples were included (Supplementary code 1).

### Normalising CRISPR/Cas9 mutation efficiency and inversion frequencies with the reference gRNA

To account for variability in transfection efficiency across all constructs, we normalised the mutation frequencies of each variable gRNA to the mutation frequency of a fixed gRNA. Both gRNAs were encoded on the same plasmid backbone and co-delivered into protoplasts in a single construct, ensuring that any variation in plasmid uptake, Cas9 expression, or gRNA transcription affected both gRNAs proportionally within a sample. The fixed gRNA targets a consistent site in all constructs, serving as a reliable internal standard for such shared variation. We calculated a normalisation factor by dividing the average mutation frequency of the fixed gRNA (calculated across all experimental samples, excluding controls) by the mutation frequency observed for the fixed gRNA in each individual sample. This normalisation factor was then applied to the mutation frequencies of the variable gRNAs and the inversion frequencies, adjusting for variability due to differences in transfection efficiency. This approach allows for an accurate comparison of the relative cutting efficiencies of different variable gRNAs, independent of extrinsic factors that could vary between experiments.

### Detecting inversions with PacBio Sequel II sequencing

For the verification of complete inversion events, DNA obtained from a separate protoplast transfection experiment was PCR-amplified using site-specific primers (Supplementary Tables 3 and 4). For each inversion size, three replicates were analysed and amplicons were prepared for PacBio SMRT-Bell sequencing following the standard protocol provided by PacBio (available at: https://www.pacb.com/wp-content/uploads/Procedure-Checklist-Preparing-SMRTbell-Libraries-using-PacBio-Barcoded-Overhang-Adapters-for-Multiplexing-Amplicons.pdf). The sequencing was performed at the Leiden University Medical Centre, The Netherlands.

Inversion events were detected in PacBio sequencing reads using the “grep” command in Linux. For each double-strand break (DSB) site within the amplicon, the search targeted a 16 bp sequence located at both the left and right sides of the DSB, spaced approximately 60 bp apart with the DSB site positioned centrally. This arrangement allows for the identification of small deletions at the DSB sites. The grep command was configured to permit random insertions ranging from 1 to 80 bp at the DSB sites, which allows for the identification of small indels around the inversion sites (Supplementary code 2).

## Results

### Demonstration of CRISPR/Cas9-induced chromosomal inversions up to 37.5 Mbp

To investigate the formation of chromosomal inversions, we used CRISPR/Cas9 to induce targeted inversions in tomato protoplasts. Because lethal chromosomal inversions can lead to underestimation of inversion frequency in certain experimental setups, we optimised our system by incubating protoplasts for 48 h post-transfection. This allowed sufficient time for mutations and chromosomal rearrangements to occur while minimising the impact of cell death due to lethal rearrangements.

**Fig. 1:**
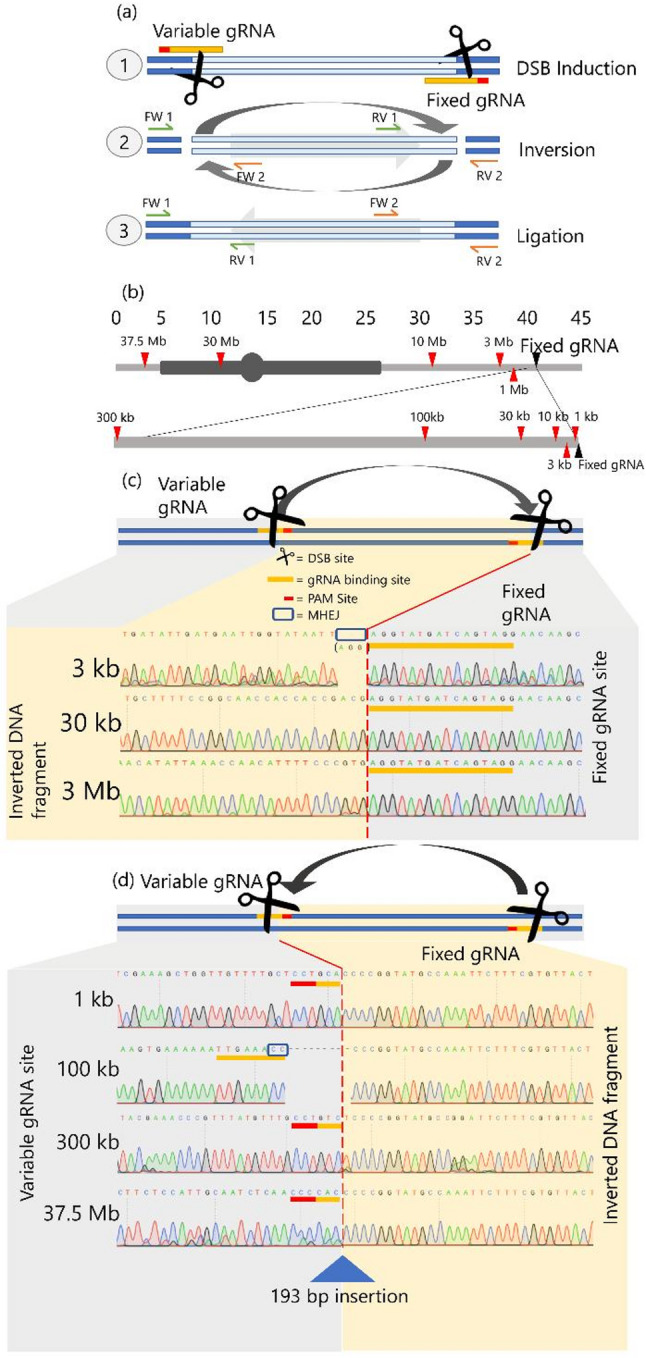
Overview showing (**a**) the gRNAs used to induce DSBs for inversion induction and the primer pairs used for amplicon generation in the case of inversion events; (**b**) depicts chromosome 6, including the fixed gRNA cutting sites (black triangle), and the variable gRNA cutting sites (red triangles), along with the distances between these DSBs. Heterochromatin and the centromere are displayed in dark grey; (**c**) Shows Sanger sequencing results of induced inversions at the fixed DSB site, with examples of 3 kbp, 30 kbp, and 3 Mbp inversions. (**d**) Sanger sequences at the ‘variable’ gRNA cutting sites, where grey and yellow areas represent the wild type DNA, and inverted DNA fragments, respectively. The expected gRNA cutting site is marked with a red-dashed line. Blue triangles indicate insertions, blue boxes indicate putative micro-homology-mediated end-joining events, yellow bars indicate gRNA binding sites, and red bars indicate PAM sites in the sequence traces. Note that in the schematic at the top of panel (**d**), the variable gRNA site is depicted as a generic block, as the specific gRNA orientation (and resulting PAM position) varies depending on the targeted inversion size. Vertical grey dotted lines indicate every tenth nucleotide

We designed 11 CRISPR/Cas9 constructs, each encoding two guide RNAs (gRNAs): a ‘fixed’ gRNA targeting a constant site on chromosome 6, and a ‘variable’ gRNA targeting one of 11 different sites along the same chromosome (Supplementary Table 1). This enabled us to generate DSBs at varying distances from the fixed site. When both DSBs were introduced simultaneously, the intervening fragment could be excised and sometimes re-integrated in an inverted orientation, resulting in a chromosomal inversion. This approach allowed us to systematically induce a broad range of inversion sizes (Fig. 1a, b).

We first confirmed successful induction of inversions in our protoplast pools using PCR with primer pairs (Supplementary Table 3) designed to amplify the predicted inversion break points (Fig. 1a). Sanger sequencing of the resulting amplicons revealed inversion events consistent with our design, including inversions as large as 37.5 Mbp (Fig. 1c, d; Supplementary Fig. 1; Supplementary Table 5). For this largest inversion of 37.5 Mbp, we detected an insertion of 193 bp at the repair site. Analysis of the sequencing data at the predicted DSB sites showed cases of precise inversion repair, while most imperfect repairs involved small deletions of three, six, or nine base pairs at the DSB site. For inversions of 3 kbp, we did not observe seamless re-ligation at the ‘fixed’ gRNA target site, but only small deletions of three or six base pairs (Fig. 1c, Supplementary Fig. 1). These small deletions likely resulted from microhomology-mediated end-joining (MHEJ; also termed MMEJ). Our ability to generate inversions across a wide size range enabled us to proceed with further experiments.

### Quantification of inversions and mutation rates using cdPCR and amplicon sequencing

**Fig. 2:**
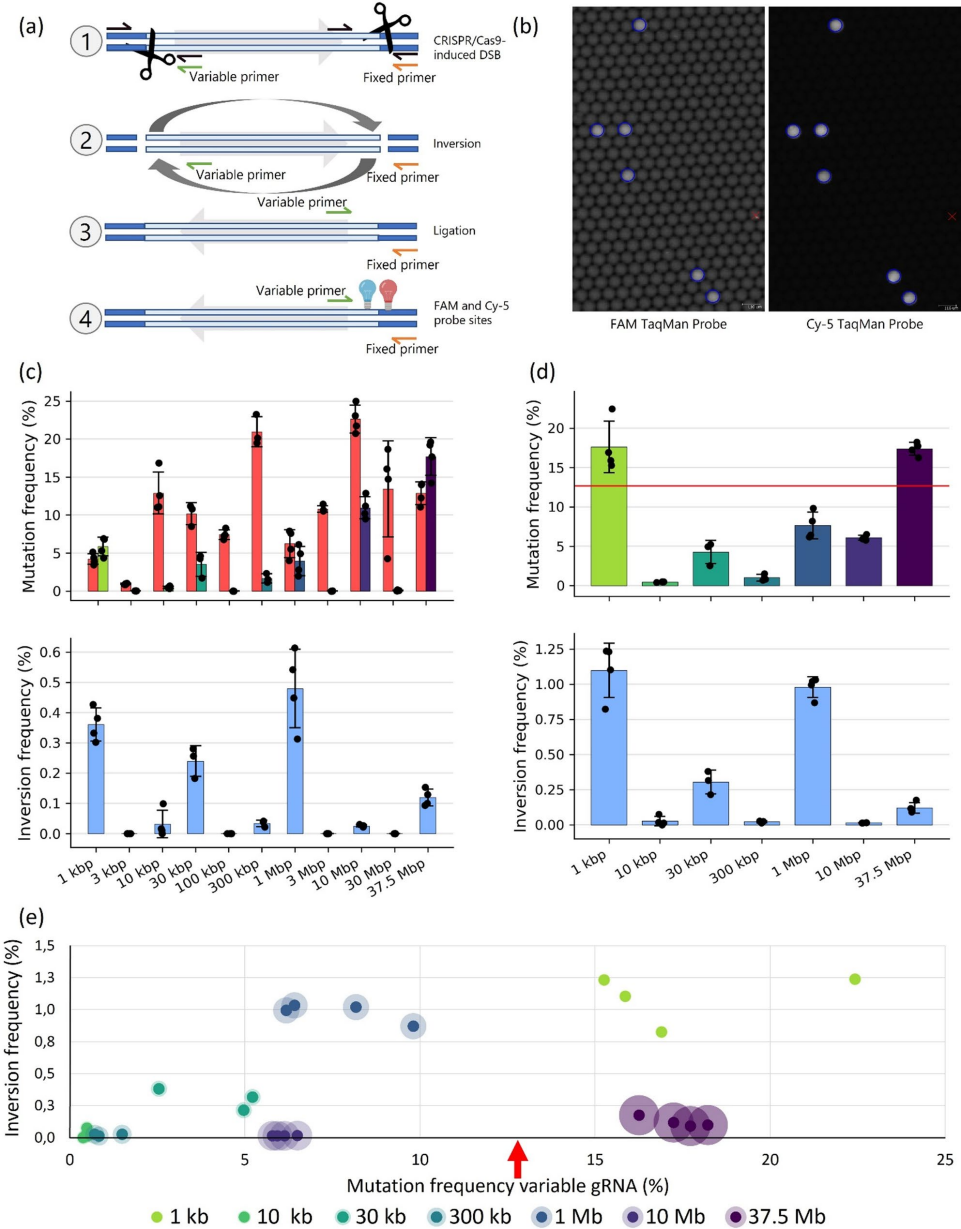
(**a**) Schematic illustration of the experimental workflow for detecting CRISPR/Cas9-induced inversions using cdPCR. Fixed and variable primer binding sites, as well as TaqMan probe positions, are indicated. Dark blue bars represent wild-type genomic regions; light blue bars indicate the region targeted for inversion. The light grey arrow shows the orientation of the to-be-inverted sequence. Scissors mark the positions of the variable (left) and fixed (right) gRNA target sites. Black arrows indicate HiSeq sequencing primer pairs used for indel quantification. The diagram illustrates the key phases of the experiment: (1) Before DSB induction, with gRNA cut sites marked by scissors; (2) After DSB induction; (3) DSB repair resulting in an inversion; (4) Binding positions of the FAM TaqMan probe (left inversion junction, blue light bulb) and Cy-5 TaqMan probe (right inversion junction, red light bulb). (**b**) High-resolution scan of partitioned reaction volumes in a cdPCR analysis of a sample containing 1 Mb inversions. Each grey dot represents a reaction volume, with inversion-positive volumes encircled in blue. The left panel shows inversions detected in the FAM channel, and the right panel shows those detected in the Cy-5 channel. Red crosses indicate reaction volumes excluded from analysis by the cdPCR Crystal Miner software. (**c**) Bar plots showing non-normalised mutation frequencies (top) and non-normalised inversion frequencies (bottom) across all tested inversion sizes. Mutation frequencies at the fixed gRNA site are shown in red, while those at the variable gRNA sites are displayed in colour (gradient from green for 1 kbp to purple for 37.5 Mbp). Mutation frequencies were determined by HiSeq sequencing; inversion frequencies were quantified by cdPCR. Dots represent individual replicates, and error bars indicate the standard deviation of four replicates, except for the 30 kbp and 300 kbp samples, which have three replicates. (**d**) Bar plots showing normalised mutation frequencies at the variable gRNA target site (top) and normalised inversion frequencies (bottom) for all samples with detectable inversions. Frequencies were normalised using the fixed gRNA as an internal reference, as described in the Methods. The horizontal red line in the top panel indicates the average fixed gRNA mutation frequency across all samples (12.74%). Samples lacking detectable inversion events were excluded. Dots represent individual replicates; error bars indicate the standard deviation. (**e**) Scatter plot showing the relationship between normalised inversion frequency (y-axis) and normalised mutation frequency at the variable gRNA site (x-axis), using the same normalised data as shown in panel D. Each point represents an individual sample replicate, colour-coded by inversion size (1 kbp to 37.5 Mbp). The transparent halo around each point indicates the size of the induced inversion. The red arrow marks the average fixed gRNA mutation frequency across all samples (12.74%)

To determine how inversion size affects its induction frequency, we set up additional protoplast transfection experiments. We divided each tomato protoplast sample transfected with a dual-gRNA CRISPR/Cas construct into two aliquots. One aliquot was used to quantify inversion events by crystal digital polymerase chain reaction (cdPCR) with inversion-specific primers (Fig. 2a; coloured primers). The other aliquot was used to measure CRISPR/Cas9-induced DSB frequencies at the ‘fixed’ and ‘variable’ gRNA target sites by amplicon sequencing (Fig. 2a; black primers). By integrating these analyses, we were able to directly compare the frequency of CRISPR/Cas9-induced inversions to the mutation rates at both break sites.

DSBs were efficiently induced at most of the predicted CRISPR/Cas9 target sites, and inversions were detected for seven distinct sizes: 1 kbp, 10 kbp, 30 kbp, 300 kbp, 1 Mbp, 10 Mbp, and 37.5 Mbp (Fig. 2c). Analysis of mutation frequencies at both the ‘fixed’ and ‘variable’ CRISPR/Cas9 target sites revealed substantial variation in gRNA cutting efficiency. While the ‘fixed’ gRNA generally exhibited high activity, the efficiency of the ‘variable’ gRNAs differed considerably. Variable gRNAs targeting the 1 kbp and 37.5 Mbp sites induced higher mutation rates than the ‘fixed’ gRNA, whereas those targeting 3 kbp, 100 kbp, 3 Mbp, and 30 Mbp displayed little or no detectable activity, as evidenced by the absence of indels at these sites (Fig. 2c). Consequently, data for these four targets were excluded from Fig. 2d and e. Correspondingly, inversions were not detected for the 3 kbp, 100 kbp, 3 Mbp, and 30 Mbp sizes, most likely due to the poor performance of the associated variable gRNAs in efficiently inducing DSBs with the CRISPR/Cas9 system. Notably, inversions were only observed when both gRNAs effectively generated DSBs; no inversions were detected in samples with poorly performing gRNAs or in negative controls (Supplementary Tables 6 and 7).

### Normalisation reduces technical variability in inversion frequency quantification

Across replicates, mutation frequencies at the ‘fixed’ gRNA site varied considerably (Fig. 2c, Supplementary Table 6), likely reflecting differences in transfection efficiency, a common challenge in protoplast transfection studies [27, 28]. In our experimental design, each construct encoded a ‘fixed’ gRNA targeting the same locus in every sample and a ‘variable’ gRNA targeting different positions. Thus, variation at the fixed site most likely results from technical differences between samples, whereas the variable site captures both technical and sequence-dependent effects. Without correcting for technical variability, genuine differences in gRNA activity or inversion efficiency would be obscured.

To address this, we developed a normalisation method using the fixed gRNA as an internal reference. Both the ‘fixed’ and ‘variable’ gRNAs were encoded on the same plasmid and co-delivered (Supplementary Fig. 3), so differences in transfection or expression affected both proportionally. We calculated a normalisation factor by dividing the average ‘fixed’ gRNA mutation frequency (across all samples) by the value in each sample and applied this to the variable gRNA mutation frequencies and inversion frequencies (Fig. 2d). This approach effectively minimised sample-to-sample variation, allowing accurate assessment of the effects of gRNA cutting efficiency, inversion size, and other variables on inversion induction.

Consistent mutation frequency ratios between the fixed and variable gRNAs were observed across replicates for each construct, even though absolute mutation frequencies varied between samples (Supplementary Fig. 2). This consistency suggests that both gRNAs are similarly affected by technical variability within each experiment, and that the relative efficiency between the fixed and variable gRNAs remains stable across samples. Thus, the fixed gRNA can serve as a robust internal control for normalising sample-to-sample variation. Additionally, applying this normalisation reduced the relative variation in measured mutation frequencies, further supporting the effectiveness of this strategy (Fig. 2c, d).

### Inversion frequency up to 1 Mbp correlates with gRNA activity in the tested intervals in chromosome 6

Having established a robust normalisation strategy, we next examined inversion frequencies and their determinants using the normalised data. We found that the frequency of chromosomal inversions was influenced by both gRNA activity and inversion size. Inversions of 1 kbp were present in 1.1% of genomes, while larger inversions of 1 Mbp occurred in 0.98% of the genomes. Inversions of 30 kbp were detected in 0.3% of the genomes, and the largest inversion tested, 37.5 Mbp in length, was observed in 0.1% of the genomes analysed. Inversions of 10 kbp, 300 kbp, and 10 Mbp were rare, each identified in less than 0.05% of the genomes analysed (Fig. 2d, e; Supplementary Table 6).

A strong positive trend was observed between the mutation frequency of the variable gRNA and the frequency of inversions for sizes up to 1 Mbp (Fig. 2e). For these inversion sizes, inversion frequency was strongly associated with variable-gRNA mutation frequency. However, because each inversion size corresponds to a different genomic interval, we cannot fully disentangle effects of physical distance from locus-specific genomic features. This linear relationship persisted up to a threshold of 12% mutation frequency (corresponding to the average activity of the fixed gRNA), beyond which further increases in variable gRNA activity did not raise inversion rates, indicating that the fixed gRNA became limiting. Taken together, within the tested intervals up to 1 Mbp, the data are consistent with gRNA cutting efficiency being a major determinant of inversion frequency, whereas a systematic distance-dependent decrease was not evident.

However, for the largest inversion sizes (10 Mbp and 37.5 Mbp), inversion frequencies were lower even when CRISPR/Cas9 mutation frequencies were high, suggesting that inversion size or locus-specific constraints may become limiting and impose additional constraints on the efficiency of inversion induction beyond the 1 Mbp threshold. Thus, while our data indicate that inversion frequency was strongly associated with gRNA efficiency within the tested intervals up to 1 Mbp, larger inversions appear to be less efficiently induced, possibly due to their size. Other factors may also contribute to the reduced frequency of these large events, and further investigation will be needed to fully disentangle these influences.

### Direct evidence for complete inversion events using long-read sequencing

**Fig. 3:**
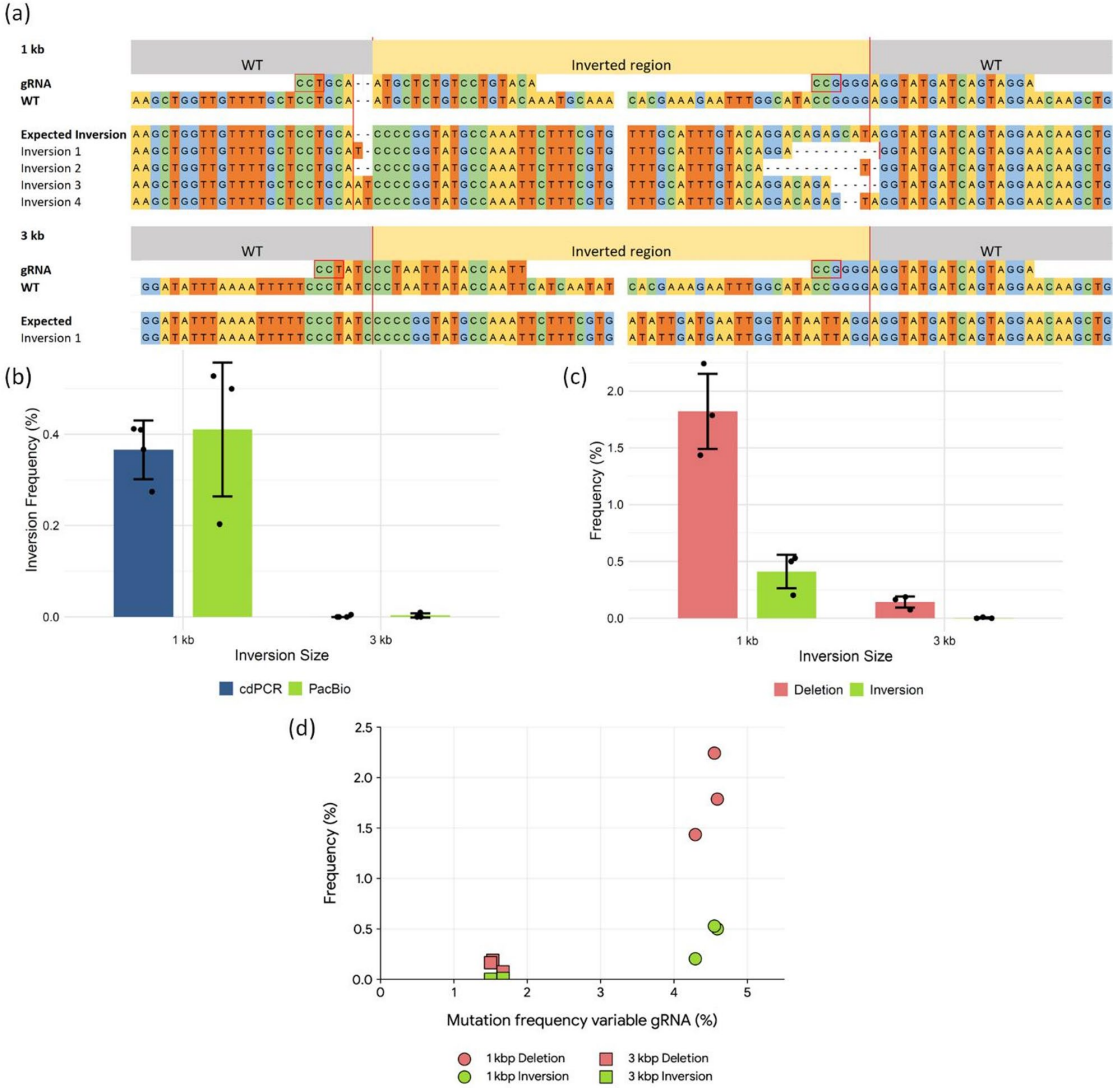
(**a**) PacBio sequencing amplicons displaying whole inversion sequences for 1 kbp and 3 kbp samples. The expected variable (left) and fixed (right) gRNA cutting sites are marked with red lines. Each inversion length shows the gRNA sequence, wild-type (WT) sequence, expected inversion sequence, and the amplicon sequence confirming inversion events. Red boxes indicate PAM sites, while grey and yellow areas represent the WT DNA segments and the inverted DNA regions, respectively. (**b**) Comparison between PacBio sequencing (green) and cdPCR + HiSeq (blue) for the detection of complete inversion events of 1 kbp and 3 kbp. The Y-axis indicates the reference-gRNA-normalised frequency of inversions. (**c**) Frequency of detected large deletions (red) and inversions (green) in the PacBio Sequencing reads for 1 kbp and 3 kbp samples. Error bars indicate standard deviations. The analysis included four replicates for cdPCR and three for PacBio sequencing. (**d**) Scatterplot displaying the relationship between the normalised mutation frequency of the variable gRNA (x-axis) and the frequency of genomic rearrangements (y-axis) for the 1 kbp and 3 kbp samples. Circles represent 1 kbp samples and squares represent 3 kbp samples. Outcomes are colour-coded as large deletions (red) and inversions (green). Each point represents a biological replicate

We recognised that our previous analyses had focused on inversion borders rather than confirming the presence of entire inversions. To address this, we conducted a third set of protoplast transfections and employed PacBio sequencing to examine the complete inversion regions in samples transfected with constructs designed to induce 1 kbp and 3 kbp inversions. This approach confirmed the successful induction of complete inversions for both sizes (Fig. 3a). Complete 1 kbp inversions were identified in 0.41% of the reads analysed, whereas inversions of 3 kbp were rare as only one unique inversion event was found (Fig. 3b, c, d). The 3 kbp inversion likely occurred at a low frequency because the ‘variable’ gRNA had low cutting efficiency at the 3 kbp site, consistent with our previous findings (Figs. 2c and 3, Supplementary Tables 8 and 9).

### High concordance between PacBio and cdPCR-HiSeq for inversion quantification

We then compared inversion frequencies determined by PacBio sequencing with those obtained using the cdPCR-HiSeq method, applying our normalisation strategy based on the ‘fixed’ gRNA as an internal standard. This allowed direct comparison between methods and experiments. Our analyses demonstrated highly consistent results between PacBio and cdPCR-HiSeq (Fig. 3b), indicating that single-border detection by cdPCR-HiSeq closely matches full-length inversion analysis by PacBio sequencing. This strong concordance reinforces the reliability of both methods for accurately quantifying inversion induction.

### Large deletions are more frequent than inversions following dual DSB induction

In addition to detecting complete inversions, our PacBio sequencing data revealed that large deletions frequently occurred in samples transfected with constructs designed to induce 1 kbp and 3 kbp inversions (Fig. 3c, d; Supplementary Tables 8 and 9). These deletions result from the excision of the DNA segment between the two DSB sites, followed by re-ligation of the DNA ends. Thus, the formation of two DSBs can yield three primary outcomes: (1) re-ligation of the broken DNA strands, with or without small indels at the DSB sites; (2) generation of an inversion; or (3) excision of the intervening region leading to a large deletion.

We next quantified the relative frequency of large deletions versus inversions. In samples transfected with the 1 kbp construct, large inter-DSB deletions occurred approximately four times more frequently than inversions (Fig. 3c, d). In the 3 kbp construct, only a single unique inversion event was detected, precluding accurate determination of the inversion-to-deletion ratio. Analysis of Fig. 3d indicates that for the 1 kbp and 3 kbp samples, the frequency of large deletions appears to reflect the same trend observed for inversions, where higher gRNA efficiency positively correlates with these types of inter-DSB rearrangements within this size range.

## Discussion

In this study, we systematically investigated how CRISPR/Cas9-induced inversion frequency in tomato depends on gRNA cutting efficiency and inversion size. By directly comparing inversion induction frequency with both mutation frequency at the CRISPR/Cas9 target sites and the physical length of the targeted chromosomal segment, we provide a quantitative assessment of these two main factors. To control for variable transfection efficiency in tomato protoplasts, we developed a normalisation approach using a reference gRNA as an internal standard. We quantified inversion events across a broad size range using cdPCR and further confirmed complete inversions by PacBio sequencing.

### Size-dependent constraints may limit CRISPR-mediated inversion induction efficiency

The most striking finding of our study is a pronounced transition in inversion frequency around 1 Mbp in our assay, beyond which CRISPR/Cas9-induced inversions were markedly less frequent. For inversions smaller than 1 Mbp, we did not observe a consistent distance-dependent decrease in induction frequency; instead, inversion frequency was strongly associated with cutting efficiency at both DSB sites. This is consistent with the requirement that inversions can only form when both breaks are generated (nearly) simultaneously, enabling the excised segment to be re-integrated in the inverted orientation.

In contrast, for inversions larger than 1 Mbp, we observed a sharp decrease in frequency even when both DSBs were efficiently induced (Fig. 2e). This suggests the existence of a previously unrecognised size-dependent barrier that limits the formation of large inversions. Although earlier studies [21, 29] reported no size-dependence for smaller inversions (up to 358 kbp), our results indicate that an additional mechanistic constraint emerges at larger genomic distances. Notably, almost all inversions induced in our study were paracentric, indicating that centromere involvement did not significantly affect inversion induction.

A limitation of our design is that inversion size is inherently linked to genomic location, because each size was generated by targeting a different variable site along chromosome 6. Therefore, differences between sizes may reflect both physical distance and locus-specific genomic features. Moreover, linear genomic distance is an imperfect proxy for spatial proximity in the nucleus. Hi-C analyses in tomato have shown higher-order chromatin organisation, including megabase-scale compartmentalisation and extensive long-range chromatin loops, which can bring loci separated by large linear distances into relatively close spatial proximity [30]. Recent high-resolution mapping of the tomato epigenome has confirmed that this chromatin folds into topologically associating domain (TAD)-like structures and compartments, often defined by specific histone modifications [31]. Consequently, the comparatively high inversion frequency observed for the 1 Mb interval could, in part, reflect locus-specific 3D contact probabilities rather than a general absence of distance effects. Testing an independent ~ 1 Mb interval at another chromosomal location would be required to assess how general the observed pattern is.

### Limited DNA end mobility may constrain large inversion formation in plants

Higher-order chromatin organisation can modulate locus-to-locus contact probability and may contribute to the comparatively high inversion frequency observed at 1 Mbp. Nevertheless, the sharp drop in inversion formation at larger separations, despite efficient induction of both DSBs, suggests that inversion generation becomes limited by distal end synapsis (i.e., the probability that the correct ends are brought into and remain in proximity for end joining) which may be constrained by locus-specific 3D folding (e.g., TAD-like organisation or insulation) and/or by limited post-break end mobility. This prompts consideration of possible additional spatial or structural constraints on DNA repair that might account for this barrier, informed by evidence from other kingdoms and guiding a mechanistic model for plants.

**Fig. 4:**
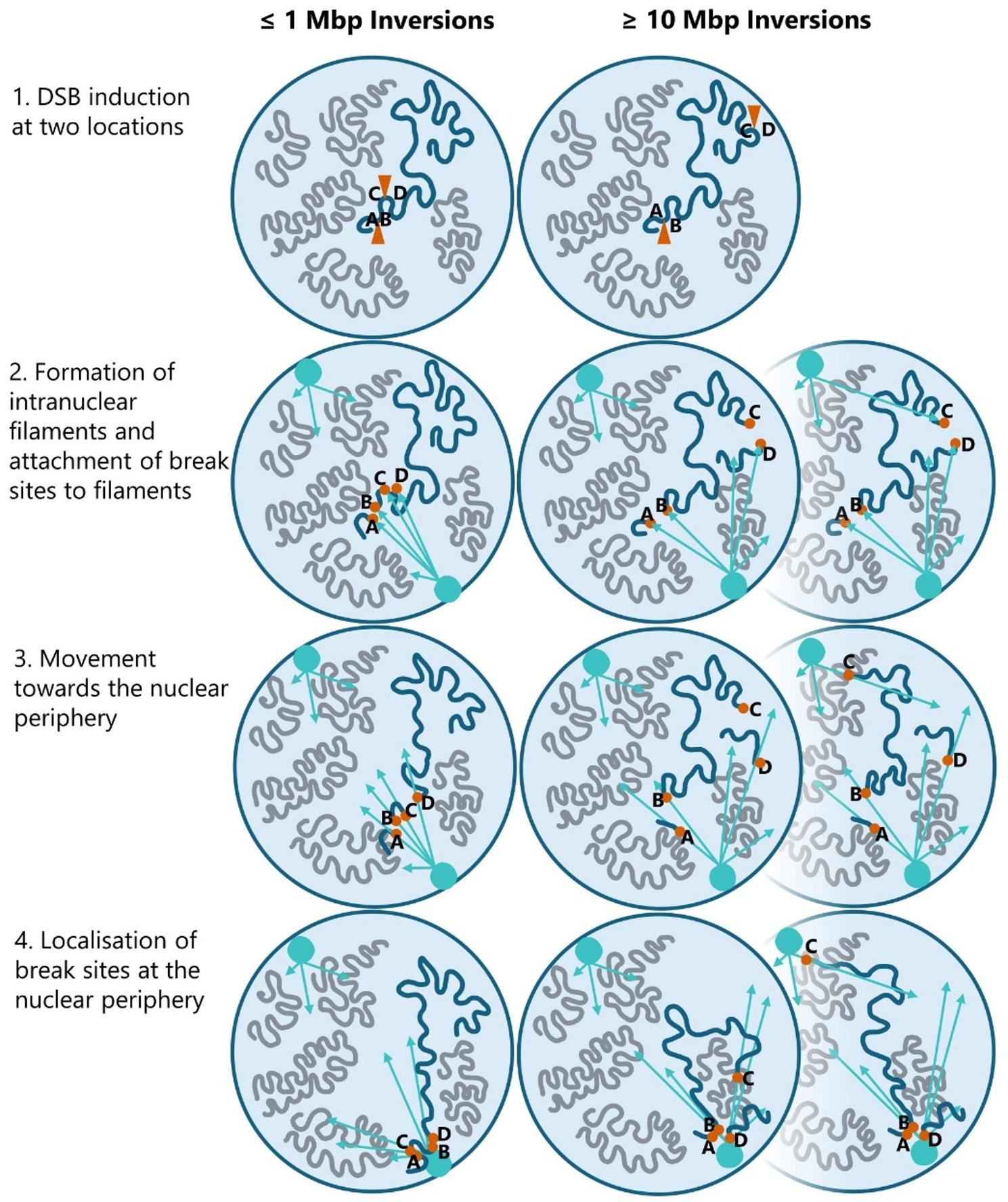
Cartoon illustration of the process of DSB migration to the nuclear periphery and repair, comparing inversions up to 1 Mbp and those equal to or larger than 10 Mbp. From top to bottom: The first panel shows the induction of DNA damage in a target chromosome (blue) with two CRISPR/Cas9-mediated DSBs, represented by orange triangles at sites A, B, C, and D. Non-targeted chromosomes are depicted in grey. For inversions ≤ 1 Mbp, the second panel illustrates the formation of DNA damage-inducible internuclear filaments extending from filament nucleation sites (cyan circles) towards the DSBs, with the targeted chromosome shown in three parts due to the DSBs. The third panel shows the active movement of the blue chromosome sections towards the nuclear periphery along the internuclear filaments (cyan arrows). The final panel shows the blue chromosome sections located at the nuclear periphery, where the DSBs come together. During the repair process, the DSBs might accidentally be ligated to the wrong ends, resulting in the formation of inversions. For inversions ≥ 10 Mbp, the second panel illustrates the formation of internuclear filaments extending from filament nucleation sites (cyan circles) towards the DSBs, with the targeted chromosome shown in three parts due to the DSBs. The third panel depicts the hampered movement of the large blue chromosome sections along the internuclear filaments due to entanglement with other nuclear structures, such as other chromosomes, and the situation where part of the chromosome (with break site C) migrates to another nucleation site. The final panel shows that not all DSB sites (A, B, C, and D) are at the nuclear periphery together, preventing their re-ligation and hampering inversion induction

Inversions typically arise from c-NHEJ-mediated repair, which is initiated by Ku70/80 complexes rapidly associating around DSBs to stabilise DNA and begin the repair process [32, 33]. In mammalian cells, c-NHEJ can be divided into rapid and slow variants [34]. The rapid variant quickly stabilises and repairs DSBs, while the slow variant, involving complex DSB processing and potential DNA segment movement to the nuclear periphery, may be employed when DSBs are complex or multiple [34, 35]. In yeast, DSBs are actively relocated to the nuclear periphery by internuclear filaments [36, 37]. Similarly, in mammalian cells, chromatin regions near DSBs exhibit increased mobility, which may facilitate the re-joining of separated DNA ends [38]. These active movements of broken DNA fragments could be important in enhancing c-NHEJ-based repair. When the two ends of a broken fragment drift apart, filament-dependent movement of DNA segments might increase the chance of reuniting and re-joining the separated DNA ends [39]. This mechanism could explain how inversions are formed when DSBs at distinct sites drift apart and are later reunited incorrectly, generating an inversion. Figure 4 illustrates this process and highlights the difference in repair efficiency between smaller inversions (≤ 1 Mbp) and larger ones (≥ 10 Mbp). Congruently, Sunder and Wilson (2019) found that the spatial proximity of CRISPR/Cas9 target sites before cleavage, whether close together or far apart, did not affect the frequency of induced inversions [29].

While such active DNA movement has been observed in mammalian and yeast cells [34, 36–38, 40], there is currently no direct evidence for similar processes in plants. Our data suggest that inducing inversions larger than 1 Mbp is more challenging, which may reflect an inefficiency in repositioning larger DNA fragments for repair. We propose that, analogous to other kingdoms, DSBs in plants that cannot be repaired by standard c-NHEJ might be relocated within the nucleus, potentially the periphery, but that the migration of large chromatin fragments may be physically constrained, reducing the efficiency of large inversion formation (Fig. 4). Although DNA in the nucleus can move relatively freely [41] and chromosomes in non-dividing cells are organised [42], the movement of large chromatin segments could still be limited.

At present, there is no direct evidence for active repositioning of large DNA fragments in plant cells following DSBs. Our hypothesis that reduced mobility and spatial constraints account for the lower frequency of large inversions in plants raises the possibility that plants possess analogous, but perhaps distinct, repair mechanisms to those described in yeast and mammals. The structural organisation of plant nuclei suggests such mechanisms could exist, even if they have not yet been directly observed.

To clarify the mechanistic basis of this phenomenon, future studies could employ live-cell imaging with fluorescent markers near CRISPR/Cas9 cut sites to visualise chromatin mobility and proximity during inversion induction. Spatial re-localisation assays such as 3D-FISH could also assess the co-localisation of DSB ends, providing insight into the constraints affecting large inversion formation in plants. A key limitation of this study is that all measurements were performed in isolated tomato protoplasts rather than in whole plants. While protoplasts provide a tractable system to quantify cutting and repair outcomes, DNA repair pathway usage, chromatin context, and nuclear organisation can differ in intact tissues that ultimately give rise to regenerated plants. In addition, although large-scale rearrangements are detectable at low single-digit percent frequencies in our cellular assay, the recovery of specific inversions in regenerated material may still be challenging because fertile tomato regeneration from protoplasts is technically demanding and often inefficient. Therefore, our conclusions should primarily be viewed as mechanistic, and the practical translation to stable, gene-edited plants and breeding applications will require validation *in planta* and/or in regenerable tissues, potentially supported by screening large populations or enrichment strategies.

### Considerations for interpreting inversion frequencies across distinct genomic intervals

For all inversions induced up to the 1 Mbp size threshold, we consistently observed a positive association between gRNA cutting efficiency and inversion frequency across the tested chromosome-6 intervals, in line with previous reports [21]. In our experiments, constructs lacking an efficient variable gRNA failed to produce inversions, indicating that inversion induction can be strongly constrained by the less effective gRNA. Given that induced inversion events are inherently influenced by gRNA efficiency, inversion frequencies cannot be interpreted independently of gRNA performance. Moreover, because each inversion size corresponds to a distinct genomic interval in our design, locus-specific features may also contribute to the observed differences between constructs. Extrapolation to other loci or experimental systems therefore requires careful validation of gRNA activity, ideally across independent genomic locations.

The dependence of inversion frequency on gRNA efficiency may be further explained by the temporal dynamics of DNA repair. The formation of an inversion or a large inter-DSB deletion typically requires the co-existence of two concurrent DSBs to release the intervening genomic segment. This co-existence relies on the first DSB remaining open until the second DSB is created. However, if the repair machinery of the cell resolves the first DSB before the second DSB is created, the two sites will never be open at the same time. In tomato protoplasts, such individual repair events are frequently precise or result in small mutations; specifically, work by Ben-Tov et al. [43] demonstrates that 30–60% of DSBs in this system are repaired with mutations that may render the target site unrecognisable to the Cas9-gRNA complex. Once a target site is mutated and “locked” by this initial repair, that locus can no longer be cleaved, which effectively precludes the possibility of future large-scale rearrangements in that cell. Consequently, the observed inversion frequency reflects only the subset of cells in which both breaks co-existed long enough for distal end joining to occur, before one break was sealed by repair that introduces a target-disrupting mutation and prevents further cleavage.

### Reference gRNA normalisation improves precision and comparability in CRISPR/Cas9 editing

The inclusion of a universally induced reference gRNA as an internal control enabled us to normalise for plasmid delivery efficiency, thereby improving the precision of CRISPR/Cas9 editing efficiency quantification. By comparing the mutation rates of variable gRNAs to those of the reference gRNA, we reduced variability arising from differences in transfection efficiency. This ensured that observed variations in editing efficiency were attributable to the intrinsic properties of the variable gRNAs, rather than to extrinsic factors (Fig. 2c, d).

Although mutation rates are an indirect proxy for Cas9 cleavage efficiency, they provide a suitable estimate, as they remain correlated with Cas9-induced DNA cleavage despite influences from repair pathways. It is important to note, however, that some DSBs may be repaired via homology-directed repair (HDR), which restores the original sequence and escapes detection as a mutation [44]. HDR is primarily active during the S and G2 phases of the cell cycle and generally plays only a minor role in leaf mesophyll-derived protoplasts, where non-homologous end joining predominates. This is consistent with recent findings by Ben-Tov et al. [43], who showed that protoplasts derived from young tomato leaves are predominantly in the G1 phase, a cell cycle stage where c-NHEJ is the primary repair mechanism. We deliberately avoided fluorescence-based measures of transfection efficiency, such as GFP expression, because they are subject to additional sources of variation including differences in detection and measurement sensitivity and potential variability in reporter gene expression that do not directly reflect gRNA or Cas9 activity. By relying on mutation rates at the target site, our approach more accurately reflects the genome editing events of interest.

Incorporating a reference gRNA during preliminary gRNA screening may aid in identifying the most effective gRNAs for subsequent, more labour-intensive stable transformation experiments. By including a reference gRNA in these initial assays, it becomes possible to directly compare the editing efficiencies of multiple gRNAs within the same experimental context. Another advantage of this approach is the refinement of off-target effect quantification. Traditional assessments often consider each gRNA in isolation, whereas normalising to a fixed reference gRNA enables more direct, unbiased comparison under identical experimental conditions, an important benefit for precision applications such as gene therapy.

However, potential drawbacks must be considered. These include the possibility of chromosomal translocations or competition among gRNAs for Cas protein binding, which could affect outcomes. To mitigate such risks, we recommend selecting a reference gRNA that targets a site on a different chromosome from the gene of interest, reducing the likelihood of interchromosomal rearrangements. Additionally, both the target and reference gRNAs should be encoded within the same construct, rather than supplied on separate plasmids, to ensure consistent delivery.

### Validation of inversion detection methods and prevalence of large deletions

We initially screened for inversions using cdPCR at a single inversion border, which could, in principle, overlook incomplete events where only one site is altered. To verify the presence of complete inversions, we followed up with PacBio sequencing, analysing the entire inversion region. Comparison of both detection methods showed highly similar results once data were normalised using our reference gRNA approach (Fig. 3b). This concordance supports the accuracy of our cdPCR approach and suggests that we neither underestimated nor overestimated inversion events. Because inversion frequencies are ultimately expressed per nuclear genome equivalent, estimates based on total extracted DNA mass could be biased upward if organellar DNA contributes substantially to the DNA pool, leading to conservative absolute inversion frequencies. Importantly, this would be unlikely to affect relative comparisons between constructs if the organellar DNA fraction is similar across samples processed with the same tissue type and extraction workflow. Notably, Schmidt et al. (2019) employed cdPCR to probe both inversion borders and reported no significant differences between detection at the two DSB sites. Collectively, these results confirm that both cdPCR and PacBio sequencing are reliable for detecting inversion events, with cdPCR offering the additional advantage of detecting large inversions beyond the range of PacBio amplicon sequencing.

Each method, however, presents distinct advantages and limitations. PacBio amplicon sequencing enables the direct identification of complete inversions, as well as other editing outcomes such as large deletions and potential chromosomal duplications, provided these events fall within the amplicon size range. This comprehensive analysis is not possible with cdPCR and HiSeq, which are restricted to detecting inversions based solely on the presence of both border sites and thus may miss alterations that disrupt primer binding, including large deletions and complex rearrangements. However, PacBio amplicon sequencing is limited by the maximum length of amplicons that can be reliably generated and sequenced, precluding analysis of very large inversion events. In contrast, cdPCR allows detection of inversions across larger genomic distances, beyond the practical limits of long-read amplicon sequencing, but cannot provide information about the molecular structure of the entire rearranged segment or detect events that lack inversion-specific primer sites. We did not observe evidence for concomitant chromosomal duplications in our experiments. However, given the inherent length constraints of PCR amplicons, potential differences in primer specificity, and additional size-selection steps during sample preparation, we cannot rule out the possibility of such duplications occurring in our transfected protoplasts. Although likely rare, such events may have occurred and represent genuine editing outcomes and their potential presence should be considered when interpreting the results.

In addition to inversions, we also identified large deletions at the DSB sites in our PacBio datasets, present in all samples targeting 1 kbp and 3 kbp inversions. For the 1 kbp construct, deletions occurred at four times the frequency of inversions, consistent with prior observations. Schmidt et al. [21] reported deletion-to-inversion ratios between 1.4 and 3.5, Zhang et al. [19] observed a ratio of 10, and Liu et al. [45] found ratios varying between 1.3 and 6.6 in *A. thaliana* and between 1.3 and 14.3 in rice. Our data thus confirm the predominance of large deletions over inversions in CRISPR/Cas9 gene editing outcomes, consistent with prior reports across multiple plant systems.

## Conclusions

In conclusion, our study provides a systematic quantification of CRISPR/Cas9-induced inversion frequencies in tomato protoplasts, focusing on the roles of gRNA cutting efficiency and inversion size. By normalising for transfection efficiency using a reference gRNA, we demonstrate that inversions from 1 kbp up to 37.5 Mbp can be detected in this transient system. For inversions up to 1 Mbp, frequency is strongly associated with the cutting efficiency at both DSB sites, rather than the physical size of the inversion itself. However, because each inversion size corresponds to a distinct genomic interval, locus-specific effects cannot be excluded and additional loci will be required to assess generality. These findings regarding generality and inversion size are relevant for the management of genomic linkage, as many inversions associated with undesirable traits fall within this size range. While this study demonstrates that the targeted induction of such inversions is achievable in a cellular context, translating these results into stable plant production remains a challenge due to the relatively low absolute frequencies reported. This challenge is further compounded by the fact that the regeneration of fertile tomato plants from protoplasts is technically demanding and often inefficient. Therefore, the practical recovery of specific inversion events would likely require large-scale screening or the development of more efficient delivery and regeneration protocols. Furthermore, we found that inversions larger than 1 Mbp were induced only rarely, even with efficient gRNAs. We suggest that this reduced efficiency is due to challenges in DSB repair, possibly relating to the active transport and mobility of large DNA segments within the nucleus, a mechanism well-documented in other kingdoms, but not yet confirmed in plants.

## Supplementary Information

Supplementary Material 1.

## Data Availability

The sequencing data generated during this study have been deposited in the Sequence Read Archive (SRA) of NCBI under BioProject PRJNA1111086. The data can be accessed at [NCBI SRA](https:/www.ncbi.nlm.nih.gov/sra) using the accession number PRJNA1111086. Data supporting the findings of this study will be openly available in accordance with FAIR (Findable, Accessible, Interoperable, and Reusable) principles.
