# Supplementary material for: Influence of gRNA efficiency and inversion size on the frequency of CRISPR/Cas9-induced chromosomal inversions in tomato protoplasts
Source: BMC Plant Biol. 2026 Mar 10;26:713. doi: 10.1186/s12870-026-08442-9 (PMC13094083; doi:10.1186/s12870-026-08442-9)
Supplement: Supplementary file 1 — Supplementary Material 1. [file 12870_2026_8442_MOESM1_ESM.docx]

Supplementary Material

**
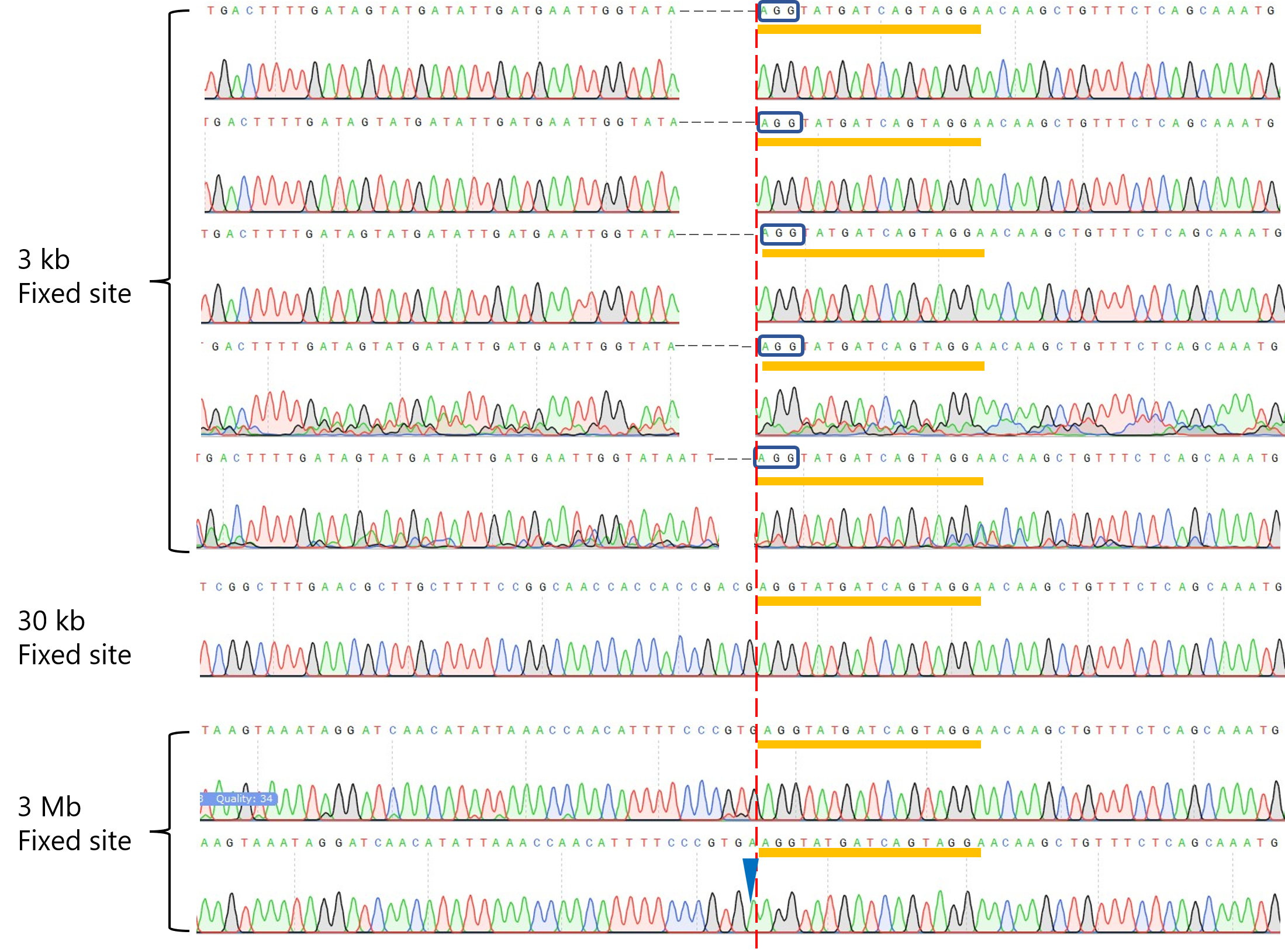
**

**
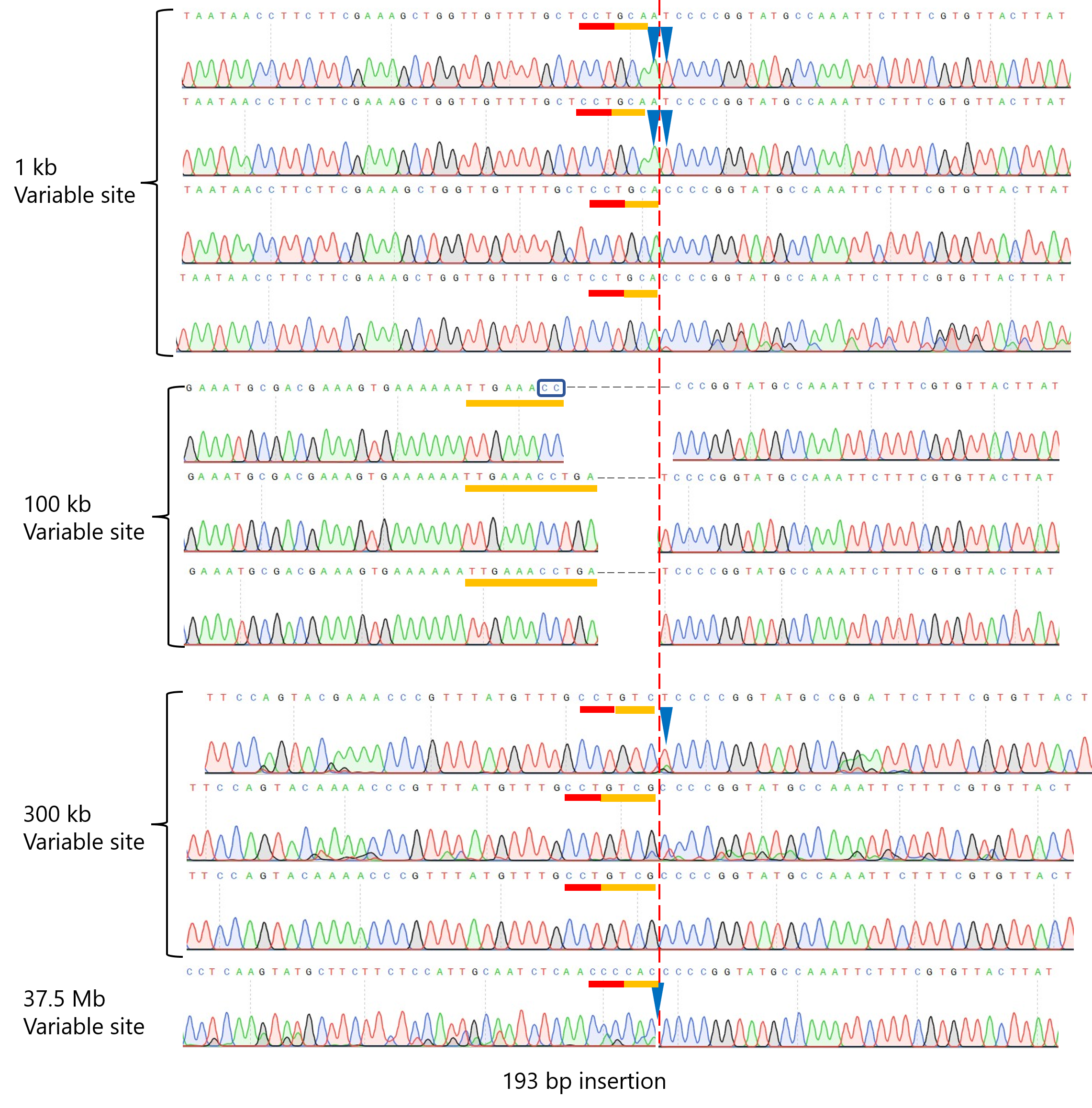
**

Supplementary Figure 1. Cartoon depicting the sequences inversion borders at either the fixed- or the variable DSB site. Dashed Red line indicates the predicted DSB border. Black vertical dashes and black triangles indicate single base-pair deletions or insertions, respectively. Blue rectangle borders indicate suspected MMEJ-based repair patterns. gRNA sequences and corresponding PAM sites are depicted as orange and red bars above the Sanger sequences, respectively.

**
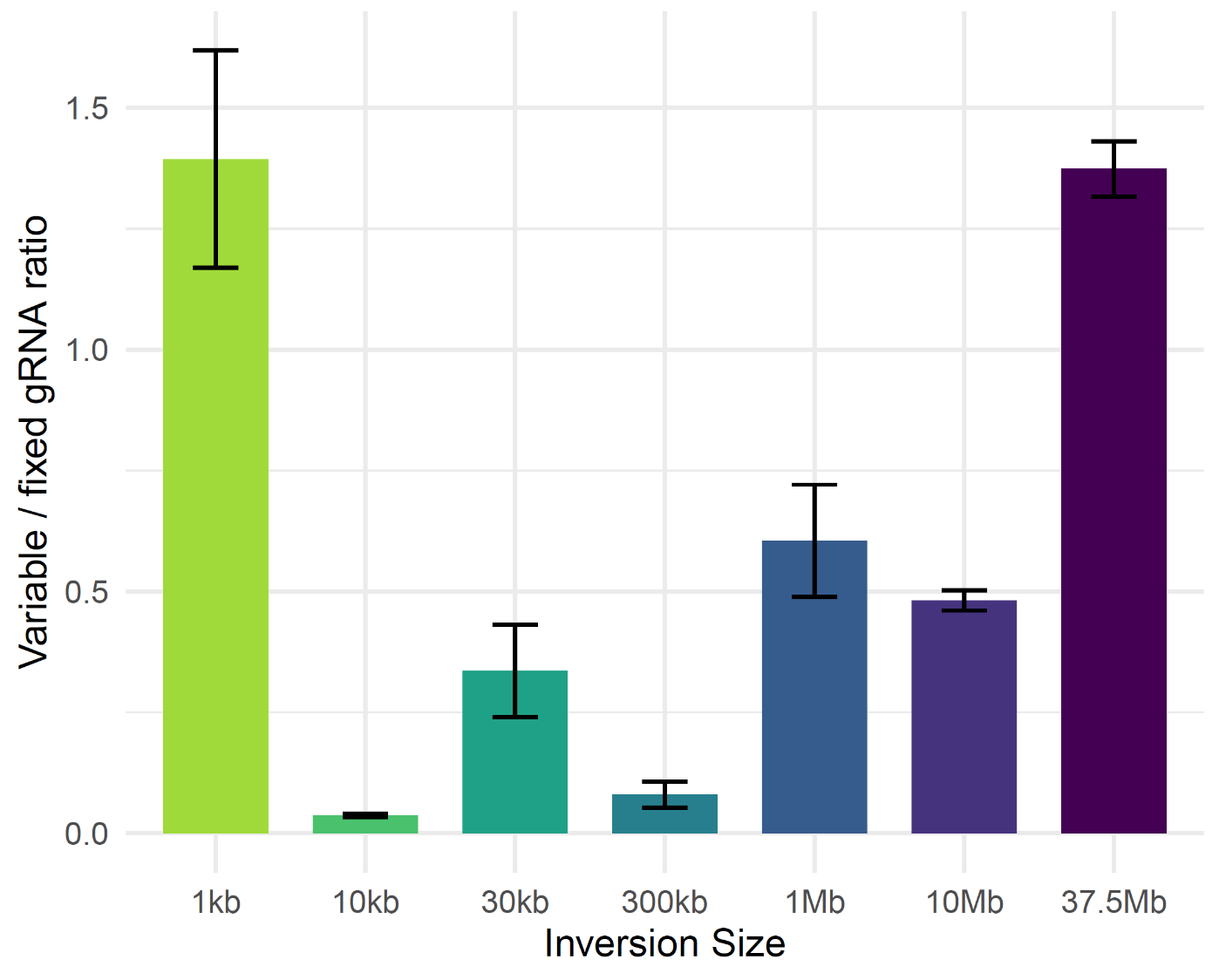
**

Supplementary Figure 2. The ratio between the ‘variable’ and the ‘fixed’ gRNA mutation induction frequency for each inversion size. Error bars represent the standard deviation from four replicates for each inversion size, except for the 30 kb and 300 kb samples. Due to a technical issue with the cdPCR chip, these samples include three replicates.


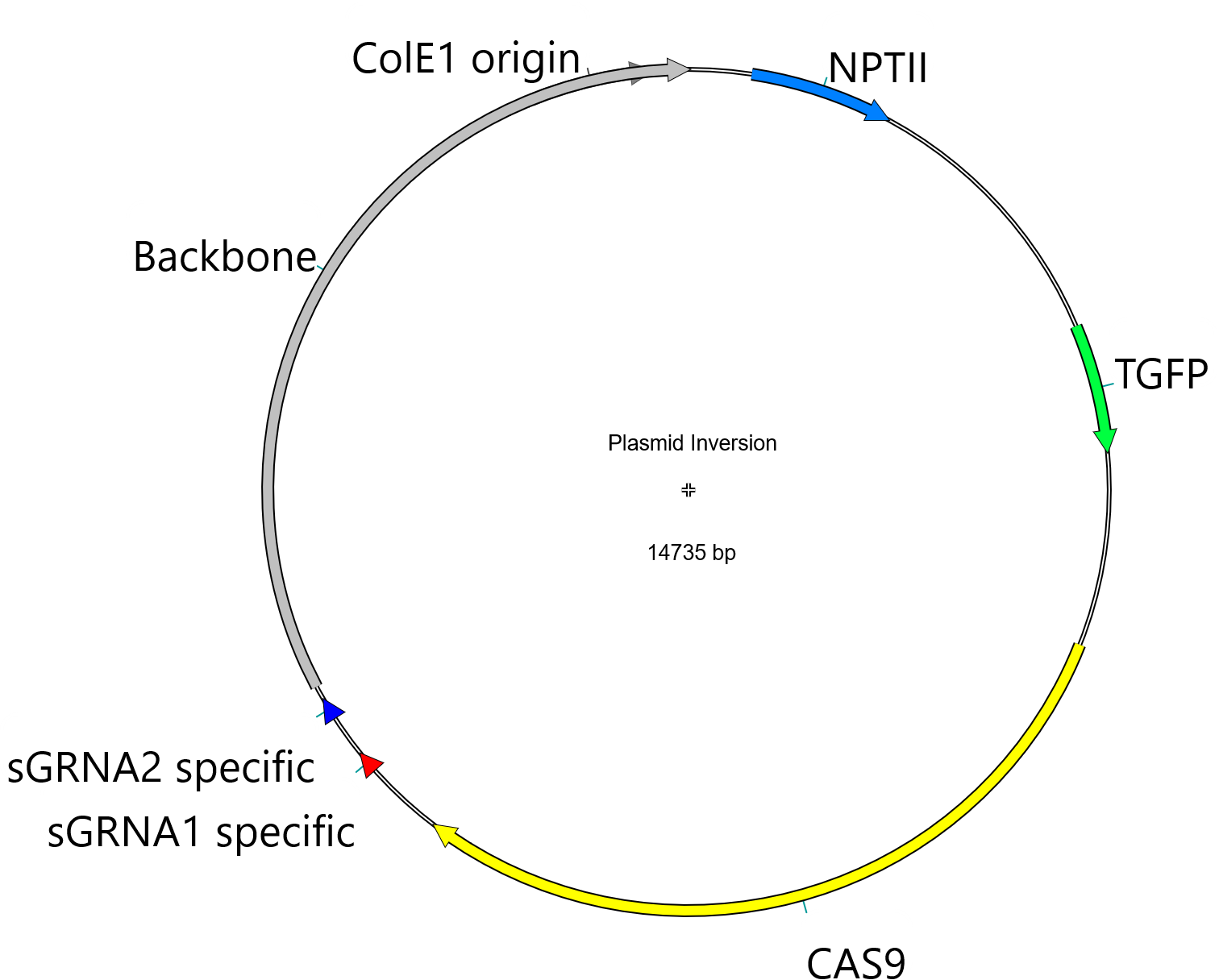


**Supplementary Figure 3.** Map of the inversion induction plasmid.

**Supplementary Table 1**

*Inversion induction gRNAs.*

| **gRNA** | **strand** | | **gRNA name** | **Distance from "Fixed gRNA" (bp)** | **Expected DSB position in SL4.0ch06** |  |
| --- | --- | --- | --- | --- | --- | --- |
| TCCTACTGATCATACCTCCC**CGG** | | - | Fixed gRNA | 0 | 40808481 – 2 | |
| TGTACAGGACAGAGCATTGC**AGG** | | - | 1 kb | 1005 | 40807476 – 7 | |
| AATTGGTATAATTAGGGAT**AGG** | | - | 3 kb | 3135 | 40805346 – 7 | |
| CAGGGAGTTGGTGTCTCCCC**TGG** | | + | 10 kb | 10184 | 40798297 – 8 | |
| TACTCCGTAACGTCCCCGT**CGG** | | + | 30 kb | 29952 | 40778529 – 0 | |
| TTGAAACCTGAGCAGCTGGG**AGG** | | + | 100 kb | 99954 | 40708527 – 8 | |
| AGTGAGTTACAGATTACGAC**AGG** | | - | 300 kb | 297704 | 40510777 – 8 | |
| TGCAATCTGCACAGGACCA**TGG** | | - | 1 Mb | 994264 | 39814217 – 8 | |
| TCATGGGTGGCATATGTCAC**GGG** | | + | 3 Mb | 2986912 | 37821569 – 0 | |
| AGATCTGTGTCGTTGCTCCA**TGG** | | + | 10 Mb | 9873024 | 30935457 – 8 | |
| AGGAACGGACTGGGCTCAAG**CGG** | | - | 30 Mb | 29395350 | 11413131 – 2 | |
| TCGTCCATCTTATGGCAGTG**GGG** | | - | 37.5 Mb | 37509359 | 3299122 – 3 | |

Supplementary Table 2

*Plasmids utilised for CRISPR-Cas9 construct generation.*

| **Plasmid content** | **Sequence (5’-> 3’)** |
| --- | --- |
| NosP::NPTII | MF375491.1 |
| pCsVMV::turboGFP | [U20341.1](https://www.ncbi.nlm.nih.gov/nucleotide/U20341.1?report=genbank&log$=nucltop&blast_rank=1&RID=R70KHK0K014) KX981585.1 |
| pUBI::Cas9 | LC906470.1 |
| Backbone | OL791271.1 |
| ColE1 | M77789.2 |

**Supplementary Table 3**

*Primer and probe names and sequences.*

| **Name** | **Sequence** |
| --- | --- |
| cdPCR_Fixed_RV | GCATGGAGAGGATACTTGAAAGA |
| cdPCR_1kb_RV | TTCCAACAAGTCTTGCAATCA |
| cdPCR_3kb_RV | ACGAATGCCAGCAAGAATTT |
| cdPCR_10kb_RV | TTAGCAGATATCAGGGGCAAT |
| cdPCR_30kb_RV | ACTTAGGTTCCTCCGGTGTG |
| cdPCR_100kb_RV | AAGAGTTGTATGGCAACTTTCAGA |
| cdPCR_Hiseq_300kb_RV_v2 | GAACTAGAAGCATCGTATGAATGG |
| cdPCR_1Mbp_RV | AAGGTTCTTTACCCGTCTGATG |
| cdPCR_3Mbp_RV | TGGAATTGTGACGTGATATGC |
| cdPCR_10Mbp_RV | TGGATGGCAATACATTAGGACAA |
| cdPCR_30Mbp_RV | TGGTACAACAAATGCAAGTAACTG |
| cdPCR_Hiseq_37.5Mbp_RV_v2 | GGATGTTCAAATCACTCTATGTGG |
| "Fixed" Probe | TCAGTAGGAACAAGCTGTTTCTCAGCA |
| 1 kb Probe | CCTGAGTCTAACACAACCTCAAAAGC |
| 10 kb Probe | TGTGAGACTAGTCACCGCTCTA |
| 30 kb Probe | CGGAAAAGCAAGCGTTCAAAGC |
| 100 kb Probe | CAAATGATTCCTCGAGGCAAACCTGC |
| 300 kb Probe | CACTTTGGTACCTTACCACTGAAACC |
| 1000 kb Probe | ATGTTTTTCTTTTCAGTGCAATCTGCAC |
| 3000 kb Probe | ATCAACATATTAAACCAACATTTTCCCG |
| 10000 kb Probe | CATCGATCTGACAATTGATTCATCAAG |
| 30000 kb Probe | CAGTCCGTTCCTTTCTCAGATGAATAAAC |
| 37500 kb Probe | CCTTTGTTTTGCTAACGTATCGTATC |
| Fixed_FW_1kb | AACCAAGGAACTTCCTCCTCAAAAACGAGA |
| Fixed_FW_3kb | AAGGTACGAACTTCCTCCTCAAAAACGAGA |
| Fixed_FW_10kb | ACCTACCTAACTTCCTCCTCAAAAACGAGA |
| Fixed_FW_30kb | ACGTGTTGAACTTCCTCCTCAAAAACGAGA |
| Fixed_FW_100kb | ACTGGACTAACTTCCTCCTCAAAAACGAGA |
| Fixed_FW_300kb | AGAGACTGAACTTCCTCCTCAAAAACGAGA |
| Fixed_FW_1Mbp | AGTCGACTAACTTCCTCCTCAAAAACGAGA |
| Fixed_FW_3Mbp | ATATGCCGAACTTCCTCCTCAAAAACGAGA |
| Fixed_FW_10Mbp | CAACCATGAACTTCCTCCTCAAAAACGAGA |
| Fixed_FW_30Mbp | CACAGTGTAACTTCCTCCTCAAAAACGAGA |
| Fixed_FW_37.5Mbp | CAGAAGTGAACTTCCTCCTCAAAAACGAGA |
| Fixed_FW_Control | CAGTGACTAACTTCCTCCTCAAAAACGAGA |
| Fixed_RV_Rep1 | AACCGGTTTATGTATGTAATTAGCATGGAGAGG |
| Fixed_RV_Rep2 | ACACACTGTATGTATGTAATTAGCATGGAGAGG |
| Fixed_RV_Rep3 | ACCTAGGTTATGTATGTAATTAGCATGGAGAGG |
| Fixed_RV_Rep4 | ACGTTGGTTATGTATGTAATTAGCATGGAGAGG |
| 1kb_FW_Treatm | ACTGTCAGCAAGTAGATGTATTTTATTTGGGGATA |
| 1kb_FW_Control | AGGAGAAGCAAGTAGATGTATTTTATTTGGGGATA |
| 3kb_FW_Treatm | ACTGTCAGACCTTTTCGAATAGTTTAGGGATATT |
| 3kb_FW_Control | AGGAGAAGACCTTTTCGAATAGTTTAGGGATATT |
| 10kb_FW_Treatm | ACTGTCAGCAGTTAACCTTCATGTTTCACTTCC |
| 10kb_FW_Control | AGGAGAAGCAGTTAACCTTCATGTTTCACTTCC |
| 30kb_FW_Treatm | ACTGTCAGTCTCAGCCACGTCACTTCTG |
| 30kb_FW_Control | AGGAGAAGTCTCAGCCACGTCACTTCTG |
| 100kb_FW_Treatm | ACTGTCAGTTGGACCGTGATCTAAAAAGTTC |
| 100kb_FW_Control | AGGAGAAGTTGGACCGTGATCTAAAAAGTTC |
| 300kb_FW_Treatm | ACTGTCAGTTGGTCTTACAAATGAAGAGAAAAAT |
| 300kb_FW_Control | AGGAGAAGTTGGTCTTACAAATGAAGAGAAAAAT |
| 1Mbp_FW_Treatm | ACTGTCAGCAGAACAATTGAAGTTGGATATCCC |
| 1Mbp_FW_Control | AGGAGAAGCAGAACAATTGAAGTTGGATATCCC |
| 3Mbp_FW_Treatm | ACTGTCAGCGAACTTCTCTATATTCACCGCTAT |
| 3Mbp_FW_Control | AGGAGAAGCGAACTTCTCTATATTCACCGCTAT |
| 10Mbp_FW_Treatm | ACTGTCAGAACTTCTCTGCTTATTGGGTTCA |
| 10Mbp_FW_Control | AGGAGAAGAACTTCTCTGCTTATTGGGTTCA |
| 30Mbp_FW_Treatm | ACTGTCAGTTCCATGAGACAAGCTTTATGGT |
| 30Mbp_FW_Control | AGGAGAAGTTCCATGAGACAAGCTTTATGGT |
| 37.5Mbp_FW_Treatm | ACTGTCAGTCTTCATTATAGTCTTCCTCACCTCA |
| 37.5Mbp_FW_Control | AGGAGAAGTCTTCATTATAGTCTTCCTCACCTCA |
| 1kb_RV_Rep1 | AACCGGTTGAGGTTGTGTTAGACTCAGGTTCA |
| 1kb_RV_Rep2 | ACACACTGGAGGTTGTGTTAGACTCAGGTTCA |
| 1kb_RV_Rep3 | ACCTAGGTGAGGTTGTGTTAGACTCAGGTTCA |
| 1kb_RV_Rep4 | ACGTTGGTGAGGTTGTGTTAGACTCAGGTTCA |
| 3kb_RV_Rep1 | AACCGGTTACGAATGCCAGCAAGAATTT |
| 3kb_RV_Rep2 | ACACACTGACGAATGCCAGCAAGAATTT |
| 3kb_RV_Rep3 | ACCTAGGTACGAATGCCAGCAAGAATTT |
| 3kb_RV_Rep4 | ACGTTGGTACGAATGCCAGCAAGAATTT |
| 10kb_RV_Rep1 | AACCGGTTGGGCAATAGTAGAGCGGTGA |
| 10kb_RV_Rep2 | ACACACTGGGGCAATAGTAGAGCGGTGA |
| 10kb_RV_Rep3 | ACCTAGGTGGGCAATAGTAGAGCGGTGA |
| 10kb_RV_Rep4 | ACGTTGGTGGGCAATAGTAGAGCGGTGA |
| 30kb_RV_Rep1 | AACCGGTTACTTAGGTTCCTCCGGTGTG |
| 30kb_RV_Rep2 | ACACACTGACTTAGGTTCCTCCGGTGTG |
| 30kb_RV_Rep3 | ACCTAGGTACTTAGGTTCCTCCGGTGTG |
| 30kb_RV_Rep4 | ACGTTGGTACTTAGGTTCCTCCGGTGTG |
| 100kb_RV_Rep1 | AACCGGTTCAACTTTCAGATATTGTCAAATGATTCC |
| 100kb_RV_Rep2 | ACACACTGCAACTTTCAGATATTGTCAAATGATTCC |
| 100kb_RV_Rep3 | ACCTAGGTCAACTTTCAGATATTGTCAAATGATTCC |
| 100kb_RV_Rep4 | ACGTTGGTCAACTTTCAGATATTGTCAAATGATTCC |
| 300kb_RV_Rep1 | AACCGGTTCATCGTATGAATGGCAAAGC |
| 300kb_RV_Rep2 | ACACACTGCATCGTATGAATGGCAAAGC |
| 300kb_RV_Rep3 | ACCTAGGTCATCGTATGAATGGCAAAGC |
| 300kb_RV_Rep4 | ACGTTGGTCATCGTATGAATGGCAAAGC |
| 1Mbp_RV_Rep1 | AACCGGTTGTTTCAAACAGATTTCATATTTCATTT |
| 1Mbp_RV_Rep2 | ACACACTGGTTTCAAACAGATTTCATATTTCATTT |
| 1Mbp_RV_Rep3 | ACCTAGGTGTTTCAAACAGATTTCATATTTCATTT |
| 1Mbp_RV_Rep4 | ACGTTGGTGTTTCAAACAGATTTCATATTTCATTT |
| 3Mbp_RV_Rep1 | AACCGGTTTGGAATTGTGACGTGATATGC |
| 3Mbp_RV_Rep2 | ACACACTGTGGAATTGTGACGTGATATGC |
| 3Mbp_RV_Rep3 | ACCTAGGTTGGAATTGTGACGTGATATGC |
| 3Mbp_RV_Rep4 | ACGTTGGTTGGAATTGTGACGTGATATGC |
| 10Mbp_RV_Rep1 | AACCGGTTTGAGAATGATGGACAATTAAGAACA |
| 10Mbp_RV_Rep2 | ACACACTGTGAGAATGATGGACAATTAAGAACA |
| 10Mbp_RV_Rep3 | ACCTAGGTTGAGAATGATGGACAATTAAGAACA |
| 10Mbp_RV_Rep4 | ACGTTGGTTGAGAATGATGGACAATTAAGAACA |
| 30Mbp_RV_Rep1 | AACCGGTTTGGTACAACAAATGCAAGTAACTG |
| 30Mbp_RV_Rep2 | ACACACTGTGGTACAACAAATGCAAGTAACTG |
| 30Mbp_RV_Rep3 | ACCTAGGTTGGTACAACAAATGCAAGTAACTG |
| 30Mbp_RV_Rep4 | ACGTTGGTTGGTACAACAAATGCAAGTAACTG |
| 37.5Mbp_RV_Rep1 | AACCGGTTCATGCCCCTCACAAATGC |
| 37.5Mbp_RV_Rep2 | ACACACTGCATGCCCCTCACAAATGC |
| 37.5Mbp_RV_Rep3 | ACCTAGGTCATGCCCCTCACAAATGC |
| 37.5Mbp_RV_Rep4 | ACGTTGGTCATGCCCCTCACAAATGC |

**Supplementary Table 4**

*gBlock names and sequences.*

| **gBlock name** | **gBlock sequence** |
| --- | --- |
| 1kbp g-block | CACCTATAGTTTCCAACAAGTCTTGCAATCAACAATTTGTTTATATCTCAATACTGTTAAGTCTTCGACTTGACCATTACAAAAGCTACTAGCTTTTGAGGTTGTGTTAGACTCAGGTTCATATCATTATATTGCATAATTGTGTATACCACCAAATTGAGTAACTTTTTGATGTTTGCATTTGTACAGGACAGAGCATAGGTATGATCAGTAGGAACAAGCTGTTTCTCAGCAAATGAGTATCTTTCATATCTTTCAAGTATCCTCTCCATGCTAATTACATA |
| 3kbp g-block | GCATTTTCTTTGTCATCTTTTCTTGATTGGTACTACCAAAAAAAAAAAATCAATTTTCAGTGGTTGTTTGGTTAAAAACAAAAATCACGAATGCCAGCAAGAATTTTCTATGCATGTAGATATAGAATATAAAAAAAAGTATATTTTTCTAAATATTTGACTTTTGATAGTATGATATTGATGAATTGGTATAATTAGGAGGTATGATCAGTAGGAACAAGCTGTTTCTCAGCAAATGAGTATCTTTCATATCTTTCAAGTATCCTCTCCATGCTAATTACATA |
| 10kbp g-block | TTACTAGAGTAGCCATCACCAAAATGTCCTAGTCCAAGGCAAAAGATTATGATAATTTTTAGTGGATAAGGAAAATGATTGCCAAAGTAGAGTAATAGACCTTATCTCCAAGATAAGCTGAACTAACCAAAACTATGTATAAAACCTTAGCAGATATCAGGGGCAATAGTAGAGCGGTGACTAGTCTCACAAACCAGGGAGGTATGATCAGTAGGAACAAGCTGTTTCTCAGCAAATGAGTATCTTTCATATCTTTCAAGTATCCTCTCCATGCTAATTACATA |
| 30kbp g-block | GGAAGAAGTTGCCGACACATCTTCTGTAGTTGCGGCGCCGGGGGTATTCGCCGTTGCTGCCGCTGCCGCTGTTGTTGTAGCAGTAAGACTGGAACTCTCACTACTAGAATTAGTATCAGACTTAGGTTCCTCCGGTGTGTCGTCGGCAGTAGTCGATTTCGGCTTTGAACGCTTGCTTTTCCGGCAACCACCACCGACGAGGTATGATCAGTAGGAACAAGCTGTTTCTCAGCAAATGAGTATCTTTCATATCTTTCAAGTATCCTCTCCATGCTAATTACATA |
| 100kbp g-block | TGACGAATAAAAGTGTTGCTAAAGCATTGTCTGGTAAAGGTGGAAATGATGCTAAATTTGGAGATTGGGGAAATTGGTTGGAGAAACATGTTGTGAATGATCAAAATTGGGATGATATTAAGAGTTGTATGGCAACTTTCAGATATTGTCAAATGATTCCTCGAGGCAAACCTGCTGATTTCTACAAATATAACCTCCCAGGTATGATCAGTAGGAACAAGCTGTTTCTCAGCAAATGAGTATCTTTCATATCTTTCAAGTATCCTCTCCATGCTAATTACATA |
| 300kbp g-block | TACAAGTCTGCCAGTCCATGCCAAACTTCATATTCATTTACCTTATCTTCCTCCACCTGTAATAGACAAATATAAAAAGTAAATAATTCAATGAAGTCAGAACTAGAAGCATCGTATGAATGGCAAAGCATATTCTGTTGTTTCACATTGAAGTTAAAAAGGTTTCAGTGGTAAGGTACCAAAGTGAGTTACAGATTACAGGTATGATCAGTAGGAACAAGCTGTTTCTCAGCAAATGAGTATCTTTCATATCTTTCAAGTATCCTCTCCATGCTAATTACATA |
| 1Mbp g-block | GTGTTTGTCTTCTTAGGTCGTATATAATGATGGTGAAACAGAGAAATTGAGGCTACATAAAGAACGATGGGAGATGCTTGAAGATAATTCAACTCAAAAGGTTCTTTACCCGTCTGATGTATACTGTTGTTTCAAACAGATTTCATATTTCATTTGTATTAGTTTTGATGTTTTTCTTTTCAGTGCAATCTGCACAGGAAGGTATGATCAGTAGGAACAAGCTGTTTCTCAGCAAATGAGTATCTTTCATATCTTTCAAGTATCCTCTCCATGCTAATTACATA |
| 3Mbp g-block | TTAATAAAATCATTTAACTATATTTATCATTAAAATTGTTTTGATATTATAAAATAAATTATTTTATGTCATATATATATATATATATATATATATATATATATATATAAGTTTAATAATAAAACTTTTGGAATTGTGACGTGATATGCTTAACTATTAAGTAAATAGGATCAACATATTAAACCAACATTTTCCCGTGAGGTATGATCAGTAGGAACAAGCTGTTTCTCAGCAAATGAGTATCTTTCATATCTTTCAAGTATCCTCTCCATGCTAATTACATA |
| 10Mbp g-block | ATAAATACAAAATTGGATGGCAATACATTAGGACAAATACGAAATCAAATATAAATATAAGCTCTTTAGTATACAAAGTGATATTGTAAAAACAAAATAAACAAGATAAATAAAAAGTTTGTTTTACATATGCAAATAGGATGGATACTTAATAATGAATACGTATTTTGGTTATATAAACTTGAGAATGATGGACAATTAAGAACATCGATCTGACAATTGATTCATCAAGTTCCATTGATTTAGTCTTTTCAGAACCATCCATGGAGGTATGATCAGTAGGAACAAGCTGTTTCTCAGCAAATGAGTATCTTTCATATCTTTCAAGTATCCTCTCCATGCTAATTACATA |
| 30Mbp g-block | AGGAAATCAAGTCTTAGATATCCTAACAATGTAAATACTTCTTGTCAATTTGCTTTGCTTGTTTCAGATCCTTTTTGTTTTGAAGAAGCGGTAGAACAATCATGATGGAAGAATGTTATGGTACAACAAATGCAAGTAACTGAAAGAAATTCTACATGGGAGTTAGTTTATTCATCTGAGAAAGGAACGGACTGGGCTCAGGTATGATCAGTAGGAACAAGCTGTTTCTCAGCAAATGAGTATCTTTCATATCTTTCAAGTATCCTCTCCATGCTAATTACATA |
| 37.5Mbp g-block | ATAGGATAACACGGTCACACATAGTTTAGACCAGTGGTGTTCACTATTATGTATCGAAACATAACTATTTTACTTACTTTTCGATAAAGGATGTTCAAATCACTCTATGTGGATATATAACACTCATGGAAATGGCATGCCCCTCACAAATGCATGCGCCTTTGTTTTGCTAACGTATCGTATCGTCCATCTTATGGCAAGGTATGATCAGTAGGAACAAGCTGTTTCTCAGCAAATGAGTATCTTTCATATCTTTCAAGTATCCTCTCCATGCTAATTACATA |

**Supplementary Table 5**

*Summary of Sanger-sequenced inversion junction repair outcomes.*

| **Inversion size** | **gRNA site** | **Repair type** | **Indel size (bp)** | **Number of times observed** | **Note** |
| --- | --- | --- | --- | --- | --- |
| 1 kb | Variable | Insertion | 2 | 2 |  |
| 1 kb | Variable | Precise repair | 0 | 2 |  |
| 3 kb | Fixed | Deletion | -3 | 2 | Microhomology-Mediated End Joining |
| 3 kb | Fixed | Deletion | -6 | 4 | Microhomology-Mediated End Joining |
| 30 kb | Fixed | Precise repair | 0 | 1 |  |
| 100 kb | Variable | Deletion | -9 | 1 | Microhomology-Mediated End Joining |
| 100 kb | Variable | Deletion | -6 | 2 |  |
| 300 kb | Variable | Insertion | 1 | 1 |  |
| 300 kb | Variable | Precise repair | 0 | 2 |  |
| 3 Mb | Fixed | Precise repair | 0 | 1 |  |
| 3 Mb | Fixed | Insertion | 1 | 1 |  |
| 37.5 Mb | Variable | Insertion | 193 | 1 | Large insertion, likely a vector component |

NOTE: Each row summarises a junction repair outcome observed by Sanger sequencing (Supplementary Figure 1), including the indel size and the number of times this outcome was observed; these counts are descriptive and not intended to estimate population-level repair frequencies in protoplast pools.

**Supplementary Table 6**

*Non-normalised frequencies of CRISPR-Cas9-induced mutations and inversions in protoplast assays.*

|  | **Variable gRNA target site** | | | **Fixed gRNA target site** | | | **Inversions** | | |
| --- | --- | --- | --- | --- | --- | --- | --- | --- | --- |
| **Sample Name** | **Analysed reads** | **Edited reads** | **Editing (%)** | **Analysed reads** | **Edited reads** | **Editing (%)** | **Genomes in analysed cdPCR volume** | **Inversion events** | **Inversion frequency (%)** |
| 1kbp_5 | 36188 | 1917 | 5,30 | 56801 | 2492 | 4,39 | 36505 | 156 | 0,43 |
| 1kbp_45 | 27304 | 1864 | 6,83 | 48668 | 2485 | 5,11 | 36385 | 121 | 0,33 |
| 1kbp_48 | 26513 | 1831 | 6,91 | 50757 | 1976 | 3,89 | 45159 | 172 | 0,38 |
| 1kbp_60 | 17247 | 749 | 4,34 | 35292 | 1221 | 3,46 | 54026 | 163 | 0,30 |
| Neg_control | 47457 | 43 | 0,09 | 45587 | 356 | 0,78 | 24157 | 0 | 0,00 |
| Neg_control | 42168 | 41 | 0,10 | 37132 | 313 | 0,84 | 24805 | 0 | 0,00 |
| 3kbp_10 | 29681 | 4 | 0,01 | 53991 | 456 | 0,84 | 41217 | 0 | 0,00 |
| 3kbp_17 | 25952 | 1 | 0,00 | 45271 | 447 | 0,99 | 31663 | 0 | 0,00 |
| 3kbp_28 | 22045 | 1 | 0,00 | 45384 | 385 | 0,85 | 42496 | 2 | 0,01 |
| 3kbp_56 | 15450 | 1 | 0,01 | 31274 | 315 | 1,01 | 43816 | 0 | 0,00 |
| Neg_control | 29681 | 4 | 0,01 | 45587 | 356 | 0,78 | 27539 | 0 | 0,00 |
| Neg_control | 25952 | 1 | 0,00 | 37132 | 313 | 0,84 | 29521 | 0 | 0,00 |
| 10kbp_26 | 47166 | 160 | 0,34 | 46546 | 5171 | 11,11 | 15019 | 0 | 0,00 |
| 10kbp_30 | 40506 | 180 | 0,44 | 38471 | 4243 | 11,03 | 18406 | 2 | 0,01 |
| 10kbp_33 | 34957 | 164 | 0,47 | 37401 | 4697 | 12,56 | 18013 | 5 | 0,03 |
| 10kbp_49 | 21171 | 140 | 0,66 | 26402 | 4452 | 16,86 | 10105 | 10 | 0,10 |
| Neg_control | 50932 | 55 | 0,11 | 45587 | 356 | 0,78 | 20226 | 0 | 0,00 |
| Neg_control | 50284 | 67 | 0,13 | 37132 | 313 | 0,84 | 23355 | 0 | 0,00 |
| 30kbp_9 | 67933 | 2885 | 4,25 | 30001 | 3242 | 10,81 | 11491 | 21 | 0,18 |
| 30kbp_15 | 45346 | 2092 | 4,61 | 26434 | 2956 | 11,18 | 9279 | 26 | 0,28 |
| 30kbp_57* | 44050 | 1157 | 2,63 | 25833 | 2358 | 9,13 | 5807 | 31 | 0,53 |
| 30kbp_58 | 29064 | 497 | 1,71 | 17110 | 1454 | 8,50 | 10148 | 26 | 0,26 |
| Neg_control | 74947 | 170 | 0,23 | 45587 | 356 | 0,78 | 35585 | 0 | 0,00 |
| Neg_control | 80252 | 170 | 0,21 | 37132 | 313 | 0,84 | 16668 | 0 | 0,00 |
| 100kbp_4 | 54588 | 0 | 0,00 | 54271 | 4005 | 7,38 | 46007 | 0 | 0,00 |
| 100kbp_53 | 65676 | 3 | 0,00 | 45873 | 3794 | 8,27 | 33168 | 0 | 0,00 |
| 100kbp_59 | 37936 | 0 | 0,00 | 45913 | 3108 | 6,77 | 36481 | 0 | 0,00 |
| 100kbp_64 | 25791 | 0 | 0,00 | 30405 | 2198 | 7,23 | 37141 | 0 | 0,00 |
| Neg_control | 122248 | 12 | 0,01 | 45587 | 356 | 0,78 | 20495 | 0 | 0,00 |
| Neg_control | 606736 | 45 | 0,01 | 37132 | 313 | 0,84 | 21590 | 0 | 0,00 |
| 300kbp_11 | 48854 | 748 | 1,53 | 60548 | 14076 | 23,25 | 69554 | 15 | 0,02 |
| 300kbp_13* | 43254 | 466 | 1,08 | 52132 | 10123 | 19,42 | 12782 | 0 | 0,00 |
| 300kbp_18 | 34904 | 394 | 1,13 | 51722 | 10416 | 20,14 | 28780 | 12 | 0,04 |
| 300kbp_37 | 12526 | 289 | 2,31 | 35415 | 6902 | 19,49 | 38529 | 15 | 0,04 |
| Neg_control | 62384 | 10 | 0,02 | 45587 | 356 | 0,78 | 7552 | 0 | 0,00 |
| Neg_control | 54163 | 7 | 0,01 | 37132 | 313 | 0,84 | 21700 | 0 | 0,00 |
| 1Mbp_6 | 39339 | 1935 | 4,92 | 69655 | 5302 | 7,61 | 32238 | 198 | 0,61 |
| 1Mbp_20 | 34180 | 954 | 2,79 | 60742 | 3340 | 5,50 | 27610 | 124 | 0,45 |
| 1Mbp_42 | 26940 | 1646 | 6,11 | 58546 | 4610 | 7,87 | 29522 | 160 | 0,54 |
| 1Mbp_62 | 21096 | 411 | 1,95 | 39998 | 1594 | 3,99 | 34774 | 109 | 0,31 |
| Neg_control | 54513 | 30 | 0,06 | 45587 | 356 | 0,78 | 22268 | 0 | 0,00 |
| Neg_control | 52487 | 9 | 0,02 | 37132 | 313 | 0,84 | 17179 | 0 | 0,00 |
| 3Mbp_3 | 50941 | 0 | 0,00 | 59022 | 6286 | 10,65 | 25746 | 0 | 0,00 |
| 3Mbp_14 | 45083 | 4 | 0,01 | 49997 | 5731 | 11,46 | 13874 | 0 | 0,00 |
| 3Mbp_47 | 38431 | 0 | 0,00 | 48909 | 5153 | 10,54 | 15272 | 0 | 0,00 |
| 3Mbp_52 | 29454 | 0 | 0,00 | 34413 | 3631 | 10,55 | 16044 | 0 | 0,00 |
| Neg_control | 61352 | 2 | 0,00 | 45587 | 356 | 0,78 | 53581 | 0 | 0,00 |
| Neg_control | 58327 | 0 | 0,00 | 37132 | 313 | 0,84 | 30511 | 0 | 0,00 |
| 10Mbp_21 | 51058 | 6563 | 12,85 | 58201 | 14550 | 25,00 | 41602 | 13 | 0,03 |
| 10Mbp_31 | 41751 | 4672 | 11,19 | 53687 | 12384 | 23,07 | 38801 | 10 | 0,03 |
| 10Mbp_43 | 36351 | 3450 | 9,49 | 52694 | 10922 | 20,73 | 55731 | 12 | 0,02 |
| 10Mbp_63 | 25131 | 2564 | 10,20 | 36951 | 8029 | 21,73 | 33783 | 8 | 0,02 |
| Neg_control | 61766 | 141 | 0,23 | 45587 | 356 | 0,78 | 40356 | 0 | 0,00 |
| Neg_control | 53605 | 93 | 0,17 | 37132 | 313 | 0,84 | 61423 | 0 | 0,00 |
| 30Mbp_22 | 262978 | 160 | 0,06 | 29465 | 4736 | 16,07 | 7389 | 0 | 0,00 |
| 30Mbp_32 | 74665 | 103 | 0,14 | 56230 | 8281 | 14,73 | 6355 | 0 | 0,00 |
| 30Mbp_36 | 48749 | 7 | 0,01 | 37847 | 1609 | 4,25 | 5018 | 0 | 0,00 |
| 30Mbp_40 | 28461 | 10 | 0,04 | 32557 | 6076 | 18,66 | 9460 | 0 | 0,00 |
| Neg_control | 77574 | 9 | 0,01 | 45587 | 356 | 0,78 | 36737 | 0 | 0,00 |
| Neg_control | 56502 | 5 | 0,01 | 37132 | 313 | 0,84 | 9704 | 0 | 0,00 |
| 37.5Mb_1 | 48308 | 6858 | 14,20 | 52454 | 5789 | 11,04 | 8501 | 13 | 0,15 |
| 37.5Mb_24 | 41446 | 8137 | 19,63 | 47758 | 6686 | 14,00 | 17953 | 18 | 0,10 |
| 37.5Mb_34 | 36900 | 6524 | 17,68 | 44418 | 5448 | 12,27 | 12776 | 12 | 0,09 |
| 37.5Mb_39 | 22326 | 4294 | 19,23 | 32235 | 4545 | 14,10 | 8442 | 11 | 0,13 |
| Neg_control | 50661 | 143 | 0,28 | 45587 | 356 | 0,78 | 54347 | 0 | 0,00 |
| Neg_control | 52100 | 175 | 0,34 | 37132 | 313 | 0,84 | 33520 | 0 | 0,00 |

NOTE*: Samples marked with an asterisk were excluded from the analysis. Sample 30kbp_57 was excluded because the total number of analysable genomes in the cdPCR chip slot was below the threshold of 6,000 genomes. Sample 300kbp_13 was excluded because the number of analysable droplets in the cdPCR chip slot was below the threshold of 18,000 droplets.

**Supplementary Table 7**

*Detection of FAM and Cy-5 positive signals and inversion events cdPCR assays.*

| **Sample name** | **Analysable droplets** | **FAM channel positive signals** | **Cy-5 channel positive signals** | **Inversion events (FAM and Cy-5 Positive)** |
| --- | --- | --- | --- | --- |
| GFP_1kbp_2 | 24499 | 0 | 0 | 0 |
| GFP_1kbp_27 | 20080 | 0 | 0 | 0 |
| Fixed_1kbp_5 | 23415 | 156 | 158 | 156 |
| Fixed_1kbp_45 | 20162 | 121 | 123 | 121 |
| Fixed_1kbp_48 | 21218 | 173 | 172 | 172 |
| Fixed_1kbp_60 | 23842 | 163 | 168 | 163 |
| MQ_control_1kbp | 20443 | 0 | 0 | 0 |
| GFP_3kbp_7 | 24108 | 2 | 0 | 0 |
| GFP_3kbp_44 | 22349 | 0 | 0 | 0 |
| Fixed_3kbp_10 | 22199 | 0 | 0 | 0 |
| Fixed_3kbp_17 | 22512 | 0 | 0 | 0 |
| Fixed_3kbp_28 | 23994 | 2 | 2 | 2 |
| Fixed_3kbp_56 | 20027 | 0 | 0 | 0 |
| MQ_control_3kbp | 25841 | 0 | 0 | 0 |
| GFP_10_kbp_12 | 21743 | 0 | 0 | 0 |
| GFP_10_kbp_46 | 22660 | 0 | 0 | 0 |
| Fixed_10kbp_26 | 23468 | 0 | 0 | 0 |
| Fixed_10kbp_30 | 23668 | 3 | 2 | 2 |
| Fixed_10kbp_33 | 23848 | 5 | 6 | 5 |
| Fixed_10kbp_49 | 21901 | 12 | 10 | 10 |
| GFP_30kbp_25 | 26566 | 0 | 0 | 0 |
| GFP_30kbp_51 | 25308 | 1 | 0 | 0 |
| Fixed_30kbp_9 | 24707 | 23 | 21 | 21 |
| Fixed_30kbp_15 | 27047 | 27 | 26 | 26 |
| Fixed_30kbp_57* | 25049 | 31 | 31 | 31 |
| Fixed_30kbp_58 | 27004 | 27 | 26 | 26 |
| MQ_control_30kbp | 26596 | 0 | 0 | 0 |
| GFP_100kbp_29 | 20786 | 0 | 0 | 0 |
| GFP_100kbp_54 | 21732 | 1 | 0 | 0 |
| Fixed_100kbp_4 | 22940 | 0 | 0 | 0 |
| Fixed_100kbp_53 | 22740 | 0 | 1 | 0 |
| Fixed_100kbp_59 | 22643 | 0 | 0 | 0 |
| Fixed_100kbp_64 | 22894 | 0 | 0 | 0 |
| MQ_control_100kbp | 21277 | 1 | 0 | 0 |
| GFP_300kbp_55 | 24873 | 0 | 0 | 0 |
| GFP_300kbp_61 | 23143 | 0 | 0 | 0 |
| Fixed_300kbp_11 | 24340 | 15 | 15 | 15 |
| Fixed_300kbp_13* | 12011 | 2 | 0 | 0 |
| Fixed_300kbp_18 | 24871 | 13 | 12 | 12 |
| Fixed_300kbp_37 | 24480 | 16 | 15 | 15 |
| MQ_control_300kbp | 27092 | 1 | 0 | 0 |
| GFP_1Mbp_19 | 21147 | 0 | 0 | 0 |
| GFP_1Mbp_38 | 19237 | 0 | 0 | 0 |
| Fixed_1Mbp_6 | 22921 | 198 | 204 | 198 |
| Fixed_1Mbp_20 | 23045 | 117 | 124 | 124 |
| Fixed_1Mbp_42 | 19737 | 160 | 165 | 160 |
| Fixed_1Mbp_62 | 23423 | 108 | 108 | 109 |
| MQ_control_1Mbp | 19228 | 0 | 0 | 0 |
| GFP_3Mbp_65 | 21177 | 0 | 0 | 0 |
| GFP_3Mbp_66 | 25868 | 0 | 0 | 0 |
| Fixed_3Mbp_3 | 21160 | 1 | 0 | 0 |
| Fixed_3Mbp_14 | 21554 | 1 | 0 | 0 |
| Fixed_3Mbp_47 | 25027 | 0 | 0 | 0 |
| Fixed_3Mbp_52 | 26293 | 0 | 0 | 0 |
| MQ_control_1Mbp | 24451 | 0 | 0 | 0 |
| GFP_10Mbp_16 | 23785 | 1 | 0 | 0 |
| GFP_10Mbp_50 | 25554 | 0 | 0 | 0 |
| Fixed_10Mbp_21 | 20255 | 13 | 13 | 13 |
| Fixed_10Mbp_31 | 25559 | 9 | 10 | 10 |
| Fixed_10Mbp_43 | 25869 | 13 | 12 | 12 |
| Fixed_10Mbp_63 | 24147 | 8 | 8 | 8 |
| MQ_control_10Mbp | 24986 | 0 | 0 | 0 |
| GFP_30_Mbp_8 | 22187 | 0 | 0 | 0 |
| GFP_30_Mbp_23 | 24375 | 0 | 0 | 0 |
| Fixed_30Mbp_36 | 20129 | 2 | 0 | 0 |
| Fixed_30Mbp_32 | 24790 | 2 | 0 | 0 |
| Fixed_30Mbp_22 | 22439 | 6 | 0 | 0 |
| Fixed_30Mbp_40 | 20504 | 2 | 0 | 0 |
| MQ_control_30Mbp | 25191 | 1 | 0 | 0 |
| GFP_37.5Mbp_35 | 26800 | 0 | 0 | 0 |
| GFP_37.5Mbp_41 | 22410 | 0 | 0 | 0 |
| Fixed_37.5Mbp_1 | 22622 | 13 | 13 | 13 |
| Fixed_37.5Mbp_24 | 21256 | 19 | 18 | 18 |
| Fixed_37.5Mbp_34 | 24181 | 13 | 12 | 12 |
| Fixed_37.5Mbp_39 | 25782 | 11 | 11 | 11 |
| MQ_control_37.5Mbp | 22972 | 0 | 0 | 0 |

NOTE*: Samples marked with an asterisk were excluded from the analysis. Sample 30kbp_57 was excluded because the total number of analysable genomes in the cdPCR chip slot was below the threshold of 6,000 genomes. Sample 300kbp_13 was excluded because the number of analysable droplets in the cdPCR chip slot was below the threshold of 18,000 droplets.

**Supplementary Table 8**

*PacBio sequencing quantification of total reads, large inter-DSB deletions, and small deletions at variable and fixed gRNA sites, including calculated fixed and variable gRNA efficiencies, correction factors, corrected variable gRNA efficiencies, and corrected large inter-DSB deletion frequencies.*

| **Sample** | **Total Reads** | **Large Inter-DSB Deletions** | **Small Deletions (Variable gRNA)** | **Small Deletions (Fixed gRNA)** | **Fixed gRNA efficiency (%)** | **Variable gRNA efficiency (%)** | **Correction factor** | **Variable gRNA efficiency corr. (%)** | **Large Inter-DSB Deletion Frequency corr. (%)** |
| --- | --- | --- | --- | --- | --- | --- | --- | --- | --- |
| 1 kbp Rep 1 | 52226 | 932 | 1077 | 1177 | 5.27 | 4.59 | 1.001 | 4.59 | 1.79 |
| 1 kbp Rep 2 | 58566 | 915 | 918 | 1185 | 5.75 | 4.67 | 0.918 | 4.29 | 1.43 |
| 1 kbp Rep 3 | 50009 | 1023 | 735 | 872 | 4.81 | 4.15 | 1.096 | 4.55 | 2.24 |
| 1 kbp Average | 53600 | 957 | 910 | 1078 | 5.28 | 4.47 | - | 4.48 | 1.82 |
| 3 kbp Rep 1 | 41461 | 87 | 108 | 1534 | 9.68 | 1.12 | 0.896 | 1.53 | 0.19 |
| 3 kbp Rep 2 | 28070 | 52 | 196 | 1103 | 9.75 | 1.11 | 0.889 | 1.5 | 0.16 |
| 3 kbp Rep 3 | 29879 | 17 | 38 | 1311 | 6.59 | 0.68 | 1.316 | 1.67 | 0.07 |
| 3 kbp Average | 33137 | 52 | 114 | 1316 | 8.68 | 0.97 | - | 1.57 | 0.14 |

**Supplementary Table 9**

*PacBio sequencing quantification of total reads, reads harbouring inversions, and inversion frequencies, including calculated fixed and variable gRNA efficiencies, correction factors, corrected variable gRNA efficiencies, and corrected inversion frequencies.*

| **Sample** | **Total Reads** | **Reads Harbouring Inversions** | **Inversion Frequency (%)** | **Fixed gRNA efficiency (%)** | **Variable gRNA efficiency (%)** | **Correction factor** | **Variable gRNA efficiency corr. (%)** | **Inversion Frequency corr. (%)** |
| --- | --- | --- | --- | --- | --- | --- | --- | --- |
| 1 kbp Rep 1 | 112978 | 566 | 0.50 | 5.27 | 4.59 | 1.001 | 4.59 | 0.50 |
| 1 kbp Rep 2 | 124119 | 275 | 0.22 | 5.75 | 4.67 | 0.918 | 4.29 | 0.20 |
| 1 kbp Rep 3 | 112548 | 542 | 0.48 | 4.81 | 4.15 | 1.096 | 4.55 | 0.53 |
| 1 kbp Average | 116548 | 461 | 0.4 | 5.28 | 4.47 | - | 4.48 | 0.41 |
| 3 kbp Rep 1 | 104550 | 0 | 0 | 9.68 | 1.12 | 0.896 | 1.53 | 0 |
| 3 kbp Rep 2 | 157424 | 0 | 0 | 9.75 | 1.11 | 0.889 | 1.5 | 0 |
| 3 kbp Rep 3 | 133982 | 10 | 0.01 | 6.59 | 0.68 | 1.316 | 1.67 | 0.01 |
| 3 kbp Average | 131985 | 3 | 0 | 8.68 | 0.97 | - | 1.57 | 0 |

**Supplementary Code**

The supplementary scripts used in this study are available at Zenodo under the DOI: 10.5281/zenodo.11509738 | https://doi.org/10.5281/zenodo.11509738.

Supplementary Code 1: This R script employs the AmpliCan package (Labun et al., 2019) to analyse CRISPR-Cas9 editing outcomes from Illumina HiSeq sequencing data. The raw .fq files are available from the NCBI Sequence Read Archive under BioProject PRJNA1111086. Analyses were performed according to the workflow described by Labun et al. (2019): “Accurate analysis of genuine CRISPR editing events with AmpliCan” (Genome Research, 29, 843–847).

Supplementary Code 2: This script detects inversion patterns at double-strand break (DSB) sites within PacBio sequencing data. To use this script, PacBio .fq files should first be converted to .fasta format. All sequencing data generated in this study are available in the NCBI Sequence Read Archive under BioProject PRJNA1111086.
